# Directed evolution of material-producing microorganisms

**DOI:** 10.1073/pnas.2403585121

**Published:** 2024-07-23

**Authors:** Julie M. Laurent, Ankit Jain, Anton Kan, Mathias Steinacher, Nadia Enrriquez Casimiro, Stavros Stavrakis, Andrew J. deMello, André R. Studart

**Affiliations:** ^a^Department of Materials, Complex Materials, ETH Zürich, Zürich 8093, Switzerland; ^b^Department of Chemistry and Applied Biosciences, Institute for Chemical and Bioengineering, ETH Zürich, Zürich 8093, Switzerland

**Keywords:** microfluidics, living materials, cellulose, directed evolution, microorganisms

## Abstract

Nature is home to a variety of microorganisms that create materials under environmentally friendly conditions. While this offers an attractive approach for sustainable manufacturing, the production of materials by native microorganisms is usually slow and synthetic biology tools to engineer faster microorganisms are only available when prior knowledge of genotype–phenotype links is available. Here, we utilize a high-throughput directed evolution platform to enhance the fitness of whole microorganisms under selection pressure and identify genetic pathways to enhance the material production capabilities of native species. Using *Komagataeibacter sucrofermentans* as a model cellulose-producing microorganism, we show that our droplet-based microfluidic platform enables the directed evolution of these bacteria toward a small number of cellulose overproducers from an initial pool of 40,000 random mutants. Sequencing of the evolved strains reveals an unexpected link between the cellulose-forming ability of the bacteria and a gene encoding a protease complex responsible for protein turnover in the cell. The ability to enhance the fitness of microorganisms toward a specific phenotype and to unravel genotype–phenotype links makes this high-throughput directed evolution platform a promising tool for the development of new strains for the sustainable manufacturing of materials.

## Significance

Microbial fabrication is a promising route for the sustainable production of materials, such as cellulose, silk, and carbonates. By harnessing material-producing species in nature or genetically modifying microbial strains, microorganisms have been used to synthesize textile, biomedical, and building materials in water and at ambient conditions. However, biofabrication is usually slow and constrained by our limited knowledge of how genes influence the material-forming ability of microorganisms. To address this, we utilized a high-throughput microfluidic technology that enabled the evolution of a cellulose-producing bacterium toward variants that synthesize 54 to 70% more material than native species. By gene-sequencing such cellulose overproducers, we found an unexpected gene–trait correlation, thus demonstrating the potential of directed evolution platforms for advancing sustainable biofabrication processes.

Microorganisms are able to produce organic and inorganic materials in water at mild temperatures using nontoxic chemicals abundantly available in nature. Examples include cellulose- and carbonate-producing bacteria (1, 2), silica-synthesizing diatoms (3), and fungi that grow chitin-rich mycelia (4, 5). By leveraging synthetic biology tools, microorganisms have also been engineered to synthesize sustainable and biocompatible protein-based materials, such as silk, collagen, and elastin (6, 7). In addition to these bio-fabrication routes, the biological activity of microorganisms has recently been utilized to produce living materials that are able to grow, self-heal, and remodel in response to environmental cues (4, 8–15). Genetically engineered living materials have also been created for catalysis, energy conversion, sensing, electronic, and biomedical applications (9, 14–24). Overall, the use of wild-type and engineered microorganisms to make materials under mild processing conditions is a compelling approach to replace current resource-demanding and energy-intensive manufacturing processes. The challenge lies in selecting microorganisms that fulfill engineering demands at the scale and speed of industrial processes.

To produce materials efficiently and at large scale, living microorganisms must display evolved traits to fulfill a biological function that matches manufacturing requirements. Soil-stabilizing bacteria that enable bio-cementation of building materials (25) and mycelia-producing fungi used for the production of sustainable leather (26) are illustrative examples in which wild-type species can be selected for a manufacturing process based on their biological function in the natural environment. Reprogramming the genome of microorganisms featuring robust metabolisms and short replication cycles, such as yeast and bacteria, is the approach used by synthetic biologists to boost production toward industrial scale. Despite the encouraging results of these strategies, a mismatch often exists between the biological function of native species and the desired manufacturing targets. When trying to engineer material-producing microorganisms, the concerted action of many genes involved in the envisioned phenotype may also complicate the process, and synthetic biology tools are still often limited to well-studied microorganisms, restricting the range of possibilites. In understudied microorganisms, the genes encoding for a specific function may be unknown, thus preventing a clear connection between genotype and phenotype. Even if a function-encoding gene is known, the fitness of the whole microorganism to an engineering setting will likely involve more than one specific trait. These challenges call for the development of other strategies for the selection of genetically programmable microorganisms for the bio-fabrication of materials. The directed evolution of whole microorganisms is a promising route to fill this gap.

Directed evolution has been widely used to improve the selectivity and activity of enzymes by exploring the vast design space available in the genome of microorganisms (27–30). Following the principles of natural selection, this approach accelerates the iterative process of genetic diversification and selection for a specific desired phenotype. Gene diversification typically occurs by inducing targeted or random mutations in the genome of the microorganism that produces the enzyme of interest (31–33). This results in a library of distinct mutants that are afterward screened based on the performance of the enzyme. Since the probability of finding a mutant with improved performance is very low, directed evolution of enzymes is often performed with an initial library containing 10^4^ to 10^7^ mutants (29, 34–36). Rare high-performance mutants can be selected from this vast pool using microfluidic approaches, which can operate at extremely high throughput if individual assays are performed inside droplets (29). While a phenotypic selection approach has previously been applied to screen magnetotactic bacteria mutants (36), high-throughput droplet-based directed evolution tools have not yet been used to select microorganisms and find apparently unconnected genotype–phenotype links useful for the bio-fabrication of materials.

Here, we utilize a droplet-based microfluidic platform for the directed evolution of cellulose-producing bacteria that can be utilized for the bio-manufacturing of sustainable and biocompatible materials. Given the broad interest in cellulose fibers as a renewable natural resource (37), the bacterium *Komagataeibacter sucrofermentans* (38–40) was chosen to illustrate the platform and to shed light on genotype–phenotype links in this microbial species. With the selection of this high-yield cellulose producer, we aimed at pushing the boundaries of a bacterial strain that is already used in state-of-the-art bio-manufacturing processes. The bacteria were evolved toward an overproducer phenotype by encapsulating, incubating, and sorting single bacteria mutants within microfluidic droplets. Bulk pellicles and engineered living materials with complex shapes were manufactured by growing films and three-dimensionally (3D) printing gels containing the evolved bacteria. Finally, the sorted overproducers were compared with the wild-type strain in terms of cellulose bulk production and genetic sequence to elucidate possible mechanisms controlling cellulose bio-synthesis in bacteria.

## Results and Discussion

**Fig. 1.**
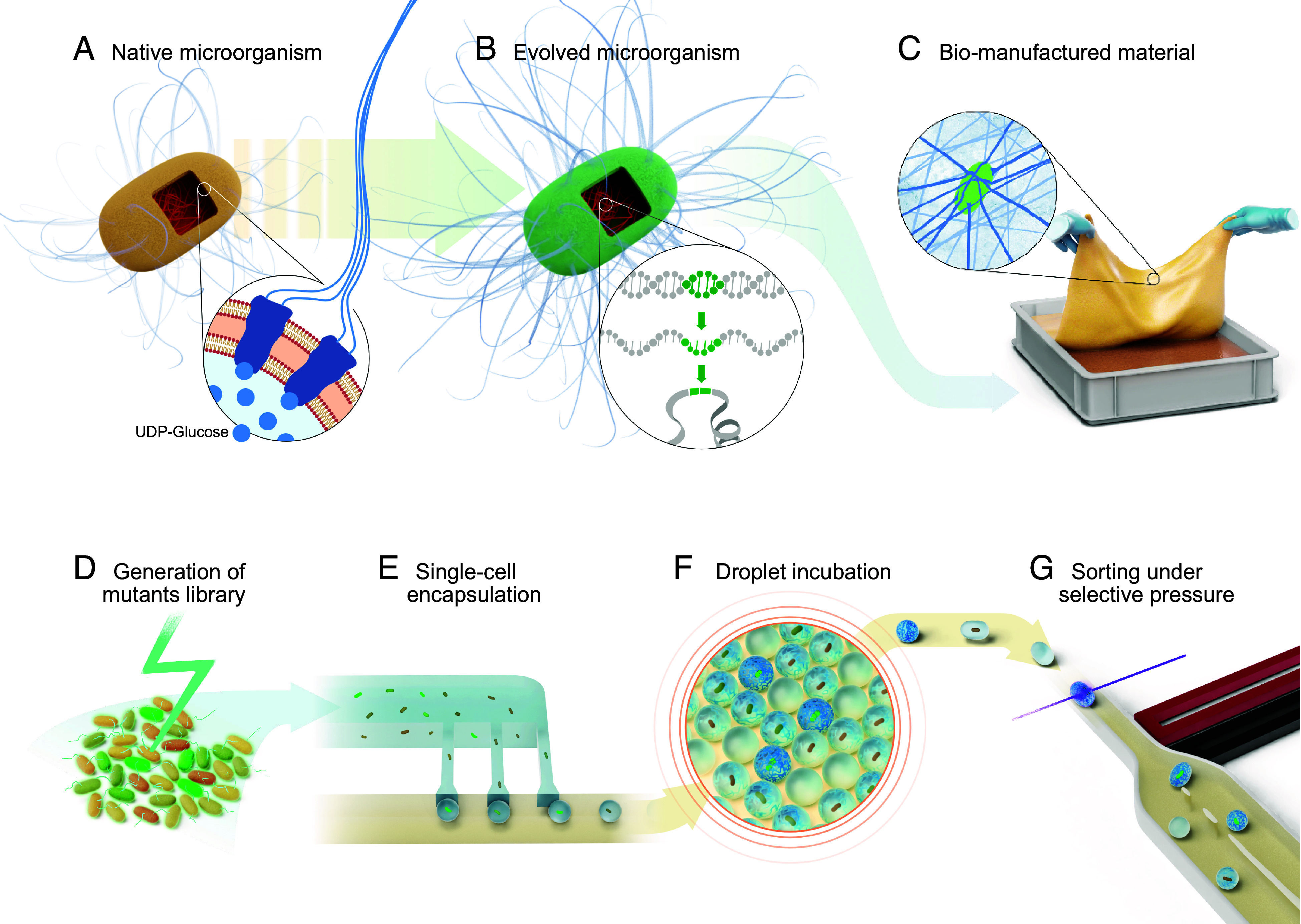
Direct evolution of cellulose-producing microorganisms for enhanced bio-fabrication. (*A*) Native *K. sucrofermentans* metabolize sugars to UDP-glucose, which is polymerized into β(1 to 4) glucan chains catalyzed by the cellulose synthase transmembrane complex. The glucan chains are exported through the cell wall and self-assembled into bundles to form nanofibers of cellulose. (*B*) Microorganisms evolved under selective pressure produce more cellulose, which can be due to mutations in their genome. Genetic mutations on a coding sequence translate into a change in the corresponding proteins, which could ultimately enhance cellulose production. (*C*) Evolved cellulose overproducers can be used for the bio-manufacturing of bulk pellicles in water at ambient temperature or for the fabrication of living materials. (*D*) The directed evolution process of a natural microorganism starts with the generation of the library of mutants using whole-genome mutagenesis. (*E*) Single-cells are encapsulated in monodisperse droplets in the presence of a cellulose-binding fluorescent dye using a high-throughput microfluidic device. (*F*) Off-chip incubation of cell-laden droplets allows for cellulose production and cell growth. (*G*) Cellulose overproducers are selected using electrically controlled dielectrophoretic forces via fluorescence-activated droplet sorting (FADS).

*K. sucrofermentans* is a gram-negative aerobic bacterium that produces long cellulose nanofibers found in food products, wound dressing materials, and high-end acoustic membranes (41). This microorganism metabolizes sugars to UDP-glucose, which is then polymerized by the transmembrane cellulose synthase complex into β(1 to 4) glucan chains that self-assemble into cellulose fibers while being exported through the cell wall (Fig. 1*A*). In the environment, the production of cellulose fibers protects the bacteria from UV radiation and harsh chemical environments (40, 42). In liquid cultures, the cellulose produced allows the microorganism to float to the air–water interface and thereby increase the access to oxygen (43).

The core cellulose synthase machinery in *K. sucrofermentans* is encoded by multiple copies of genes *bcsA*, *bcsB*, *bcsC*, and *bcsD*, alongside several accessory genes, which are present in various operons throughout the genome (44, 45). Since the function of some of those genes is not yet fully understood and that other genes are expected to be involved in the regulation of the cellulose formation process, deliberate genome editing in this microorganism is not straightforward. To circumvent this issue, we opted to use whole-genome mutagenesis in our directed evolution approach to generate variants with desirable cellulose synthesis phenotypes without prior assumptions on genotype–phenotype links.

For many established and prospective applications, the slow growth of bacteria-produced cellulose pellicles represents a challenge that prevents the broader utilization of this sustainable biopolymer. To address this issue, we established the amount of cellulose produced by the bacteria in 24 h as the target phenotype in our directed evolution process. Our hypothesis is that bacteria subjected to this selection pressure will evolve strategies to increase the throughput of their biological machinery (Fig. 1*B*). If successful, such an evolutionary process will lead to strains with genetic mutations that favor faster cellulose production. These evolved microorganisms can then be exploited to create cellulose-based macroscopic objects using state-of-the-art manufacturing technologies, such as casting, molding, and 3D printing. By combining the bottom–up cellulose-producing capability of the evolved bacteria with top–down shaping methods, we expect to bio-manufacture materials with unique multiscale architectures (Fig. 1*C*).

The directed evolution process of our cellulose-producing microorganism starts by creating a library of mutants (Fig. 1*D*). In contrast to the directed evolution of enzymes, our goal is to enhance bacterial cellulose (BC) production by exploring the entire genome of the microorganism rather than introducing mutations only in the genes encoding a specific target protein. To this end, we selected a random mutagenesis approach using Ultraviolet-C (UV-C) light to generate the mutant library. This approach allows us to tap into the broad diversity of possible genetic mutants without prior assumptions about genes that might control cellulose biosynthesis. From this initial library, we selected mutants with the desired phenotype through a series of directed evolution cycles using a droplet microfluidic platform (Fig. 1 *E*–*G*). The encapsulation of a single bacterium in individual droplets is a crucial feature of this approach, since it establishes a direct link between genotype and phenotype.

The microfluidic platform consists of three parts: a droplet generator for cell encapsulation, an incubation container for cellulose production and cell growth in droplets, and a high-speed fluorescence-activated droplet sorter (FADS) (46). To enable the directed evolution of individual cells with unique genomes, we encapsulate one microorganism per droplet by adjusting the initial cell concentration in the culture medium containing a cellulose-binding fluorescent dye. After encapsulation, cell-laden stable droplets are incubated in glass containers for up to 4 d under optimum growth conditions (*SI Appendix*, Fig. S1). Droplets are then reinjected in the FADS and those containing mutants that overproduce cellulose are dielectrophoretically separated from the rest of the population by applying an electrical pulse when the measured fluorescence exceeds a user-defined threshold. This approach relies on the fact that the amount of cellulose produced by the encapsulated bacterium is directly correlated to the measured fluorescence. Microorganisms from sorted droplets are finally compared to the native strain to identify true overproducers in larger cultures.

**Fig. 2.**
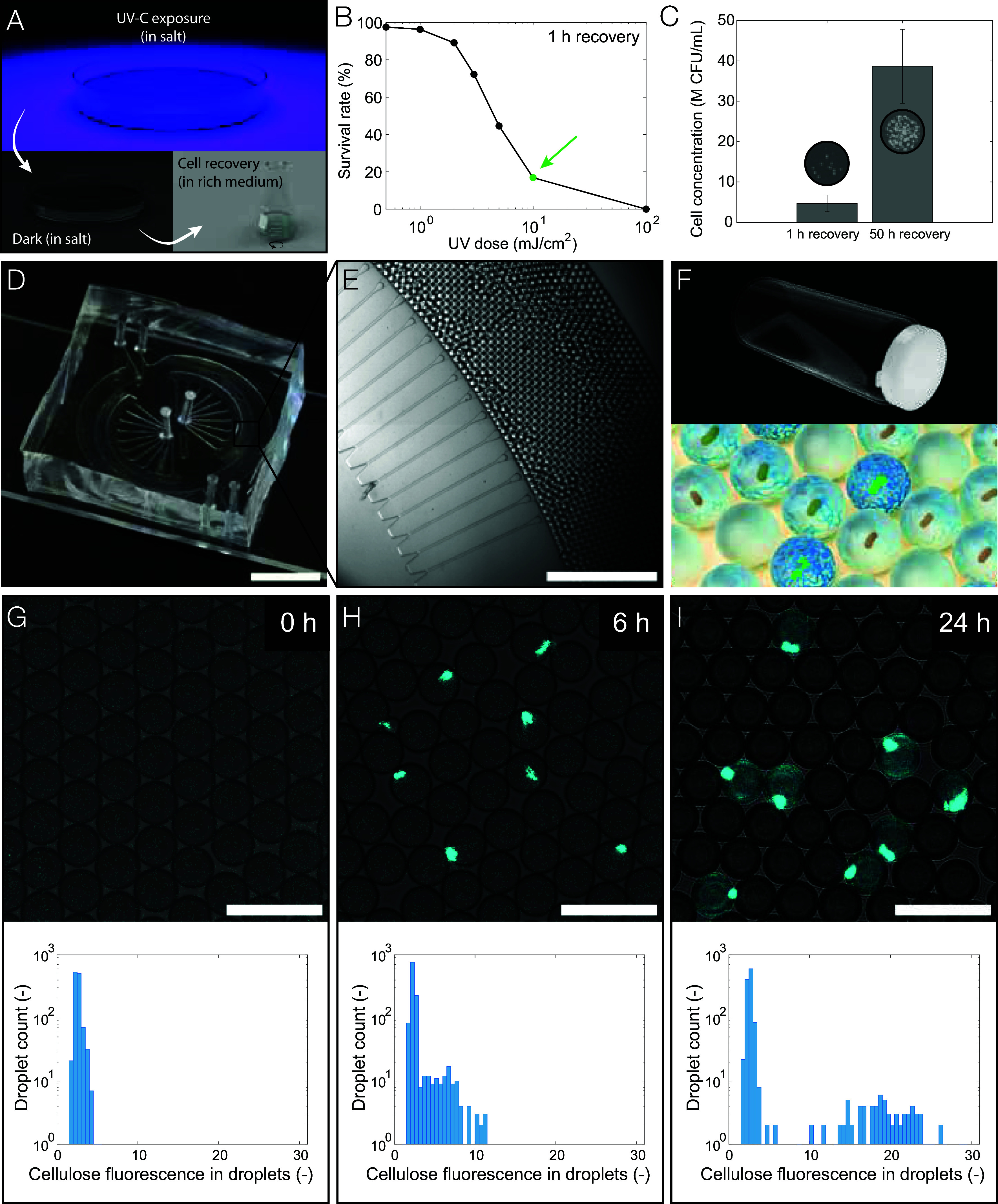
Mutagenesis, single-cell encapsulation, and BC quantification in droplets. (*A*) Mutagenesis steps used to create a mutant library. After UV-C exposure, the salt-containing cell suspension is left in the dark for 1 h. This is followed by a recovery step in rich medium for 1 h or 50 h, and finally freezing. (*B*) Survival rates of cell suspensions exposed to different UV-C doses compared to a nonexposed sample. The culture exposed to 10 mJ/cm^2^ has a survival rate of 17% and was selected for the directed evolution process (green arrow). (*C*) Impact of cell recovery step after mutagenesis to enrich the suspension with fast-growing bacteria. Error bars represent the SD. *Insets* show the CFUs from 5 µL cell suspensions at a 10^−3^ dilution after 1 and 50 h of recovery. (*D*) Step emulsification microfluidic device used to generate populations of monodisperse droplets. (Scale bar: 1 cm.) (*E*) Parallelized channels producing monodisperse droplets at a rate of 2,000 droplets per second. (Scale bar: 1 mm.) (*F*) Horizontal droplet incubation vial to ensure a droplet monolayer and therefore equal oxygen availability to encapsulated bacteria (*Top*). Over 1 d of incubation, cells grow and cellulose is produced in the droplets (*Bottom*). (*G*–*I*) Single-cell laden droplets over different incubation times (λ ~ 0.1 CFU/droplet). Native bacteria were used in these experiments to assess cellulose formation in droplets. The *Top* row displays representative confocal microscopy images, with droplets shown in gray and fluorescently labeled cellulose in cyan. (Scale bar: 100 μm.) The *Bottom* row presents corresponding histograms of cellulose-fluorescence per droplet, quantified by image analysis (*n* ~ 1,200 droplets). First, cellulose forms predominantly around the encapsulated bacteria (*H*), and then occupies most of the droplet volume (*I*).

The generation of bacterial mutants relies on the damage of DNA upon exposure of cells to UV-C irradiation. However, if DNA damage is too severe, the viability of cells and thus the directed evolution process are compromised. To create a large library of bacteria with a fraction of viable mutants, we investigated the effect of UV-C irradiation dose on the survival rate of cells (Fig. 2 *A*–*C*). Here, *K. sucrofermentans* suspended in a salt solution were irradiated with UV-C light at doses between 0.5 and 100 mJ/cm^2^. After exposure, samples were kept in the dark for 1 h to inhibit the onset of DNA repair mechanisms that prevent mutagenesis (47). This was followed by the recovery of the cells in a nutrient-rich medium for an hour (Fig. 2*A*). To quantify the cell survival rate, bacterial cultures exposed to different light doses were directly frozen for subsequent colony-forming unit (CFU) counting analysis.

The CFU counting analysis revealed that the survival rate of the irradiated bacteria decreased from 98 to 17% upon an increase in the UV-C dose from 0.5 to 10 mJ/cm^2^ (Fig. 2*B*). The survival rate was calculated relative to a control sample, which underwent the same processing but was not irradiated. Exposure to the highest dose of 100 mJ/cm^2^ led to severe DNA damage and complete cell death. Based on these results, a dose of 10 mJ/cm^2^ was found to be most appropriate to ensure a high fraction of mutants in the population while keeping bacteria viable. Cells exposed to 10 mJ/cm^2^ were further recovered for 50 h in rich culture medium as a primary selection step to enrich the suspension with fast-growing bacteria. During this period, the concentration of mutant bacteria was found to increase by eightfold from 4.7 to 38.7 million CFU/mL (Fig. 2*C*). This relatively concentrated cell suspension was frozen for later use as feedstock for the microfluidic encapsulation process.

Mutant and native bacteria were encapsulated in 49-μm droplets using a microfluidic device via the parallelized step emulsification approach (Fig. 2 *D* and *E*). Operation at a throughput of thousands of droplets per second and a droplet polydispersity index of only 10^−3^ allows the generation of large libraries of mutants with tightly controlled growth conditions that are ideal for directed evolution experiments. In the emulsification process, droplets of the diluted bacteria suspension are emulsified in a fluorocarbon oil and stabilized by a biocompatible surfactant. The number of cells encapsulated in a single microfluidic droplet is known to follow Poisson statistics and depends on the concentration of bacteria in the feedstock suspension (48). We thus tuned the bacteria suspension concentration (λ ~ 0.1 CFU/droplet) to generate a droplet population with ~90.5% of them being empty, ~9.0% containing a single cell, and ~0.5% containing more than one cell (*SI Appendix*, Fig. S2) (29). In this way, cell-laden droplets should almost exclusively contain a single genome, coupling the genotype and phenotype.

A critical aspect of droplet-based directed evolution processes is to design an assay for fast on-chip quantification of the performance of encapsulated mutants. Leveraging the ease and availability of fluorescent-based detection tools, we used Fluorescent Brightener 28 (FB) to quantify the production of cellulose by encapsulated bacteria. This commercially available dye is known to selectively bind to the (1 to 4)β bonds present in cellulose. To detect cellulose formation inside the droplets during incubation, we introduced the dye directly into the bacteria suspension used in the encapsulation process. The cellulose-forming capabilities of the encapsulated bacteria were measured using confocal microscopy after incubating the monodisperse droplets at 28 °C for periods up to 4 d (Fig. 2*F* and *SI Appendix*, Fig. S2).

Confocal microscopy images acquired during the incubation period show that 6 h were already sufficient for bacteria to start producing cellulose inside droplets. This indicates that the confinement in droplets does not stop the microorganism from synthesizing cellulose. At this early stage, the cellulose predominantly formed around the encapsulated bacteria. After extending the incubation period to 24 h, the cellulose produced was no longer confined to the vicinity of the bacteria but occupied most of the droplet volume. Fluorescence distribution histograms obtained by image analysis clearly show the emergence of a population of cellulose-containing droplets after 6 and 24 h of incubation (Fig. 2 *G*–*I*). These results validate the effectiveness of the fluorescence-based approach to quantify the cellulose-producing capabilities of the bacteria encapsulated in droplets.

**Fig. 3.**
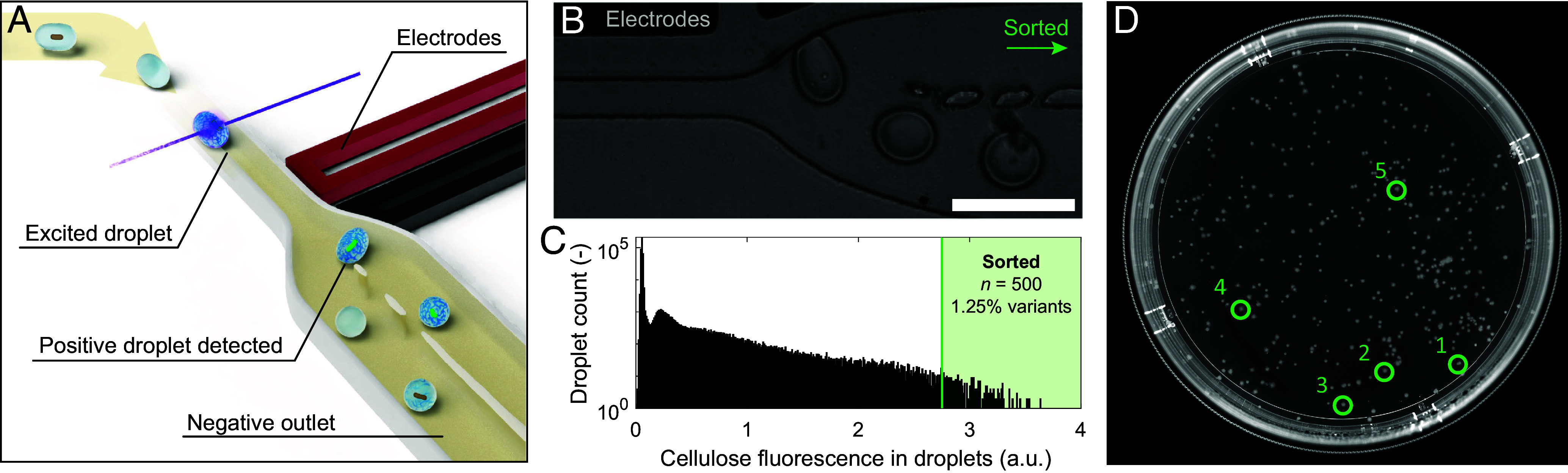
Fluorescence-activated droplet sorting and cultivation of evolved strains. (*A*) Schematics depicting the droplet sorting process. After 24 h of incubation, cell-laden (and empty) droplets pass through a laser, which excites the cellulose-bound fluorescent dye. If the PMT detector records a voltage higher than a user-defined threshold, the corresponding droplet is sorted dielectrophoretically. (*B*) Image displaying the microfluidic sorter in action. (Scale bar: 100 μm.) (*C*) Histogram of the cellulose-fluorescence signals in droplets containing mutated cells (10 mJ/cm^2^, 50 h recovery) after incubation for 24 h (λ ~0.1 CFU/droplet, 430,000 droplet events, ~40,000 mutants screened). Droplets with a cellulose-fluorescence signal corresponding to a PMT voltage above 2.75 (green box) were sorted. The sorted droplets correspond to 0.12% of all droplets and ~1.25% of all mutant-laden droplets. (*D*) Grown colonies of selected mutants after 4 d. Sorted droplets containing evolved bacteria were collected and spread onto solid medium to enable the growth of individual mutants into colonies. Five of them were chosen for further analysis (Ev1 to Ev5, green circles).

Due to the high emulsification rate of 2,000 droplets per second, the microfluidic platform enabled the generation of a library of 1.2 million droplets in only 10 min. This corresponds to approximately 100,000 single-cell encapsulated mutants. A fraction of this large pool of mutants (430,000 droplets, ~40,000 mutants) was screened in 9 min in the microfluidic droplet sorter to select for potential cellulose overproducers after 24 h of droplet incubation off-chip (800 Hz). In such a FADS device, the flowing droplets are excited using a laser, and the emitted fluorescence is detected by a photomultiplier tube (PMT; Fig. 3*A*) (29). The PMT voltage signal is used as a proxy for the fluorescence quantification in droplets. In addition to the cellulose-binding fluorescent dye, fluorescein was added in the droplets to serve as a baseline detection for all droplets.

Droplets with fluorescence above a user-defined threshold corresponding to cellulose overproducers are dielectrophoretically pulled into a separate outlet on the chip, while the vast majority is discarded in a waste outlet (Fig. 3 *A* and *B*). Preliminary experiments showed the importance of using a recovery time of 50 h after UV-C exposure to obtain a strong cellulose-fluorescence signal after 1 d of bacteria incubation in droplets (*SI Appendix*, Fig. S3). For droplets loaded with bacteria obtained after the 50-h recovery process, the PMT voltage threshold was set to 2.75 V to separate the top 1.25% most fluorescent mutants from the pool (Fig. 3*C*). Sorted droplets containing evolved bacteria were collected and spread on solid medium to enable the growth of individual mutants into colonies (Fig. 3*D*). Five colonies of evolved bacteria (Ev1 to Ev5) were arbitrarily chosen for further analysis.

**Fig. 4.**
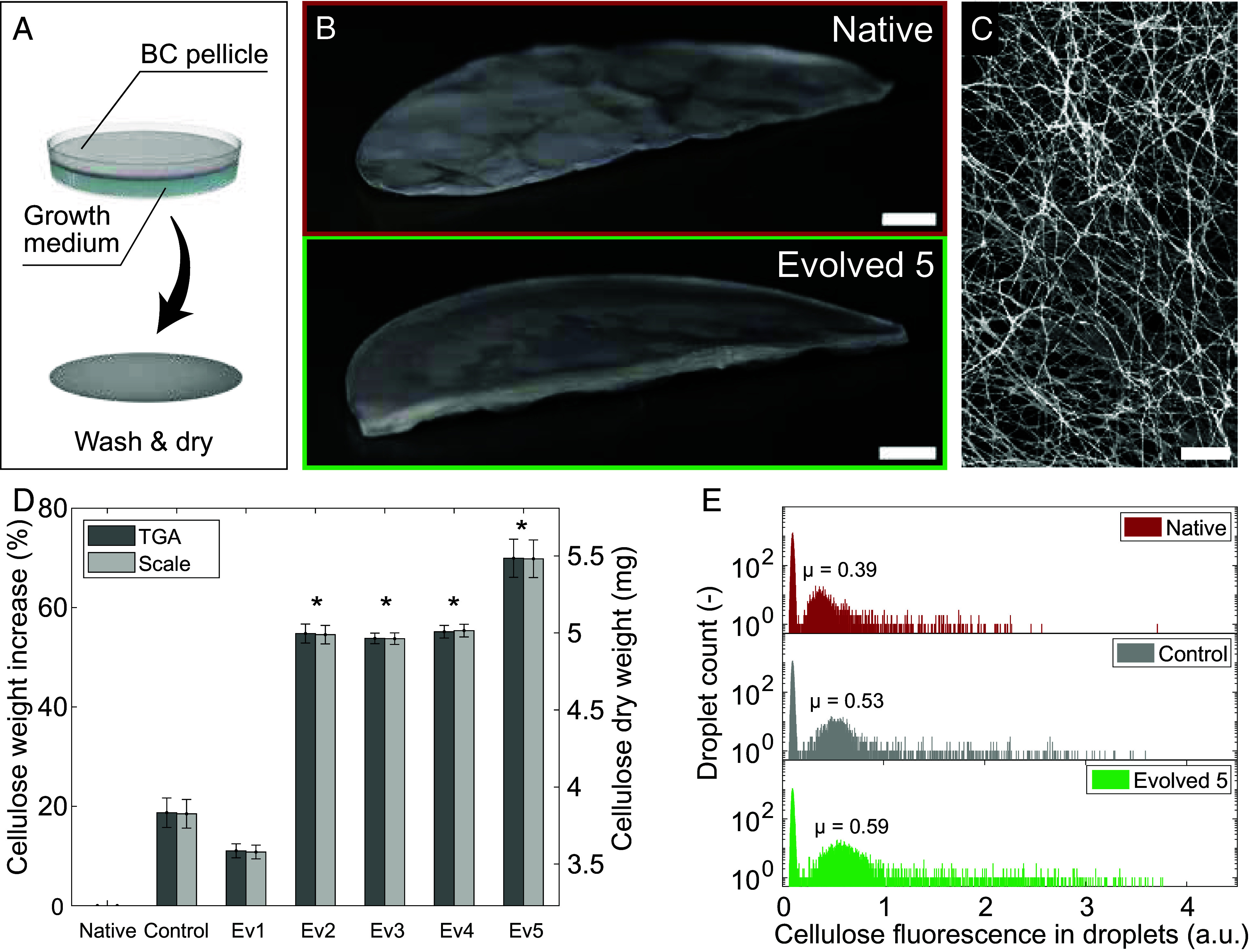
Cellulose-producing capability of evolved strains. (*A*) For the formation of bulk materials, BC pellicles are grown from single colonies in medium for 12 d, washed, and dried. (*B*) Pictures of washed and freeze-dried BC pellicles (Ø = 9.65 cm, 100 mL 2× growth medium). (Scale bar: 1 cm.) (*C*) Scanning electron microscopy image of the network of cellulose fibers synthesized by the evolved five strain. (Scale bar: 2 μm.) (*D*) Weight measurements of washed and air-dried BC pellicles grown for 12 d from single colonies (Ø = 3 cm, 5 mL growth medium). Weights were measured with both a laboratory scale and via TGA. Error bars correspond to the propagated error for the percentage data. Evolved strains (Ev2 to Ev5) showed significantly increased cellulose production (54 to 70%) compared to the native strain (*P < 0.05, *n* = 3). (*E*) Histograms of cellulose-fluorescence signal in droplets containing native, control, or evolved cells (λ ~ 0.1 CFU/droplet, 15,000 droplet events). The fluorescence peaks were fitted with a Gaussian distribution, leading to mean cellulose-fluorescence values (μ) of 0.388, 0.527, and 0.590 for the native, control, and evolved bacteria, respectively.

The evolved bacteria were compared to the native and control (0 mJ/cm^2^) strains by measuring their cellulose-forming capabilities inside droplets or in bulk pellicles. For the comparison of bulk pellicles, we cultivated the evolved, native, and control microorganisms from single colonies in liquid culture that allows for the growth of a BC pellicle at the air–water interface in static conditions (Fig. 4*A*). A long incubation time of 12 d was chosen to ensure that cells reached the stationary growth phase and to maximize cellulose production. This also minimized the possible effect of slight differences in the initial inoculation amounts (*SI Appendix*, Fig. S4). Pellicles were then washed and dried to quantify the amount of cellulose produced by the different variants by visual inspection, weight measurements, and thermogravimetric analysis (TGA).

Visual inspection of freeze-dried pellicles clearly shows the production of thicker pellicles by the evolved bacteria compared to the native species (Fig. 4*B*). Both scale measurements and TGA of air-dried pellicles heated up to 650 °C (*SI Appendix*, Fig. S5) revealed that four of the five evolved strains chosen for analysis produced 54 to 70% more cellulose compared to the native bacteria (Fig. 4*D*). The long fibers obtained from these strains show the typical interwoven microstructure of BC (Fig. 4*C* and *SI Appendix*, Fig. S6). Interestingly, the nonsorted control strain also showed a 19% increase in cellulose production relative to the native species (Fig. 4*D*), suggesting that the stresses imposed on the bacteria during the treatments for mutagenesis (exposure to salt, darkness, and the recovery process) can promote cellulose formation. The production of more extracellular polymers in response to stress is a common protective strategy observed in biofilm-forming microorganisms. These results indicate that mutants selected for fast cellulose production in droplets also led to bulk pellicles with the highest total cellulose mass after 12 d of incubation. The fact that the overproducer was obtained through a naturally occurring mutagenesis process makes the strain attractive for industrial applications, since it is not categorized as a genetically modified organism.

In addition to the bulk pellicles, the overproduction of cellulose by the evolved bacteria was also confirmed by fluorescence measurements in droplets. For analysis in droplets, bacteria were re-encapsulated (λ ~ 0.1 CFU/droplet), incubated for 24 h, and screened using the same microfluidic platform used for the initial evolution experiment. This time, no sorting was performed. Instead, we focused on the analysis of the fluorescence intensity distributions obtained for the native, control, and evolved bacteria. Evolved mutants clearly outperformed the native and control bacteria in terms of cellulose production inside droplets. Fluorescence intensity histograms obtained for these three variants show a Gaussian-like distribution that is strongly skewed toward high fluorescence values (Fig. 4*E*). To quantify the performance of the distinct bacteria, we fitted the distributions to a Gaussian mixture function, taking into account the baseline corresponding to cell-empty droplets. The mean fluorescence intensity of the peak corresponding to droplets containing cellulose could thus be compared between strains. The control and evolved bacteria displayed a mean cellulose-fluorescence 36% and 52%, respectively, higher than that measured for the native strain. This enhanced cellulose-producing capability is also evidenced by higher maximum fluorescence signals from droplets loaded with evolved bacteria. Indeed, the percentage of variants with a fluorescence higher than 3 V was 0.07%, 0.84%, and 2.46% for the native, control, and evolved samples, respectively.

To evaluate the stability of the enhanced phenotype, we performed passage experiments in which BC pellicles were grown from previous pellicles over multiple generations. The results showed that the higher production of cellulose by the selected evolved strain (Ev5) was stable even after 5 passages (*SI Appendix*, Fig. S7). Besides this stability experiment, we also performed an additional directed evolution cycle of the Ev5 strain to explore the possibility of further phenotype improvement. To this end, the evolved strain was mutated, encapsulated, incubated, and sorted with two different thresholds: the same as in the first round (2.75 V) and a more stringent one (3.60 V). The selected strains of this second round of directed evolution did not show a further increase in cellulose production but maintained a similar production as in the first evolution round (*SI Appendix*, Fig. S8). Additional mutations leading to further cellulose production increase might be possible, but it would require more directed evolution cycles.

In addition to forming thick bulk pellicles, the ability of the evolved bacteria to overproduce cellulose also opens new opportunities for the manufacturing of complex-shaped engineered living materials. To demonstrate this, we prepared a 3D printable gel that can be loaded with the evolved cellulose-producing bacteria (*SI Appendix*, Fig. S9 *A*–*C*). Network-forming silica particles, hyaluronic acid, and κ-carrageenan were used to tune the rheological properties of the gel (10). Cell-laden gels were successfully printed into a three-dimensional object with complex geometry on the centimeter scale (*SI Appendix*, Fig. S9*B*). In addition to rheology modifiers, the ink also contained nutrients required for the proliferation and growth of the embedded bacteria. This allowed for the in situ production of cellulose fibers within the printed gel during an incubation period of 1 d. The results revealed that the evolved bacteria produced a distinct pattern with more cellulose than the native bacteria also within the printable ink (*SI Appendix*, Fig. S9 *E*–*H*). This example illustrates how the architecture of engineered living materials can be finely tuned at various length scales by combining the top–down manufacturing capabilities of 3D printing with the bottom–up self-assembly processes controlled by different strains of microorganisms.

**Fig. 5.**
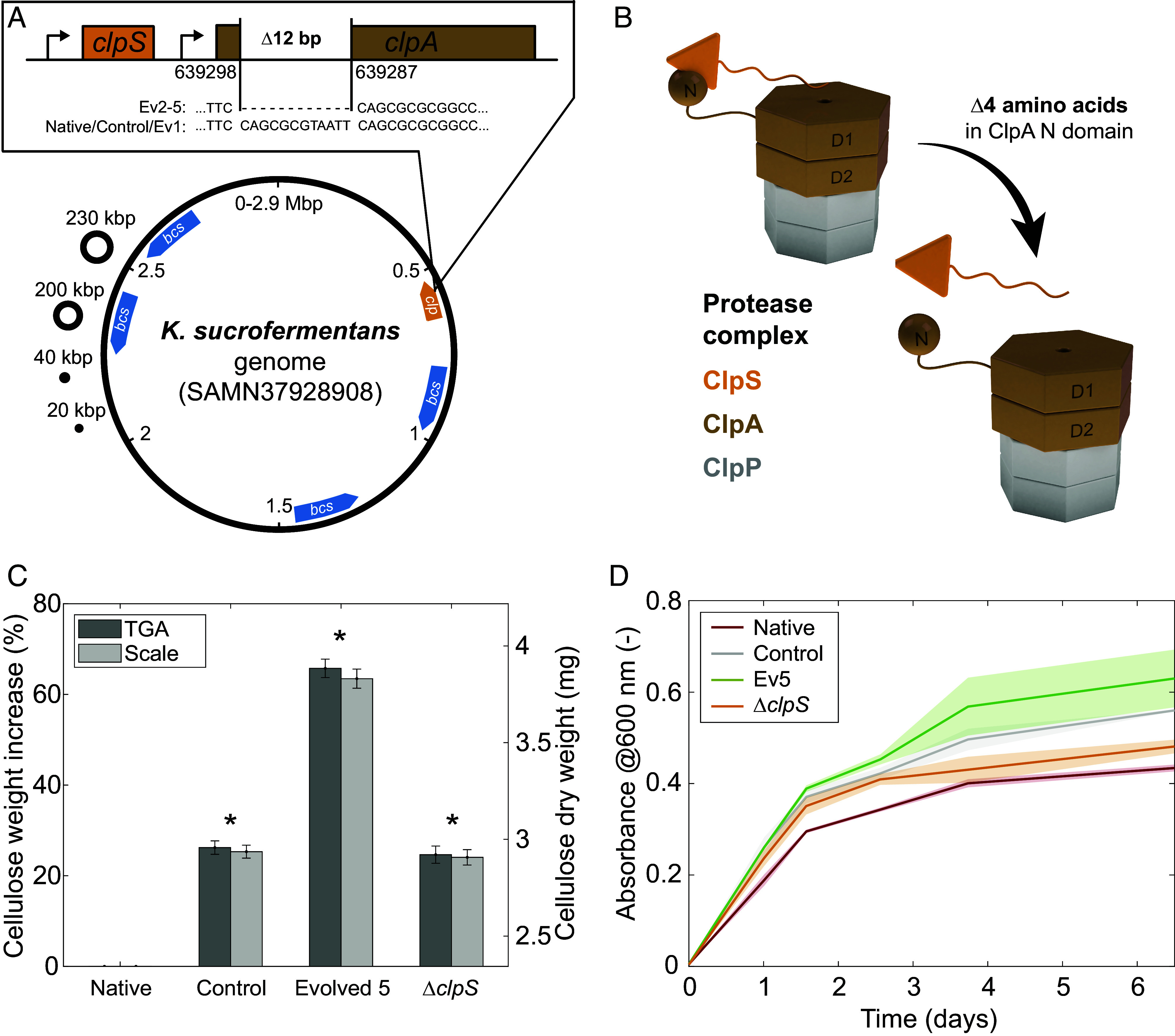
Genomic analysis and mechanistic validation of gene–trait link. (*A*) Genome representation of the native strain (SAMN37928908, not to scale) (50). Compared to the native bacteria, the evolved strains Ev2 to Ev5 display a 12-bp deletion in *clpA*, a gene coding for a protein involved in the ClpAPS protease complex. (*B*) At the protein level, the genomic deletion translates into a four-amino acid deletion in ClpA at the binding site for ClpS. Assuming that this ClpS–ClpA binding is impacted by the deletion, a *ΔclpS* knockout strain was engineered to validate this mechanism. The cartoon was adapted from Kim et al. (51) (*C*) TGA and scale weight measurements of washed and air-dried BC pellicles (Ø = 3 cm, 5 mL growth medium) produced by the native, evolved (Ev5), and *ΔclpS* knockout strains after 12 d of incubation from single colonies. Error bars correspond to the propagated error for the percentage data. The Control, Evolved 5, and *ΔclpS* knockout strains showed cellulose production 26%, 66%, and 25% higher compared to the native strain, respectively. Statistical analysis indicates a significantly different amount of cellulose produced by all the strains compared to the native strain (*P < 0.05, *n* = 5). (*D*) Bacterial growth curves of the different strains in the presence of 2 vol% cellulase in shaking conditions. Shaded areas around the curves represent the SD (*n* = 3).

The biological machinery that controls the self-assembly of cellulose fibers in *K. sucrofermentans* can be affected by mutations in the bacterial genome or by metabolic changes arising from environmental stresses. To elucidate the origin of enhanced cellulose production in the evolved strains, we compared the genome of the evolved bacteria with that of the native strain. For this, a high-quality reference genome was obtained, using both shotgun and long-read methods to sequence the native strain. The assembled genome contained a single 2.95 Mbp genome containing four separate operons with cellulose synthase genes, and four plasmids (Fig. 5*A* and *SI Appendix*, Fig. S12), with a mean read depth of 112× (*SI Appendix*, Table S1). The genomes of the evolved and control strains were sequenced with the Illumina platform and aligned to the reference genome. The breseq algorithm (49) was used to predict mutations from annotated sequences.

Genomic analysis revealed no mutations in the control strain, whereas the four evolved strains identified as cellulose overproducers (Ev2 to Ev5) shared a consistent and unique mutation: a 12-base pair deletion within the reading frame of the *clpA* gene (Fig. 5*A*). Surprisingly, the deleted base pairs are not part of the *bcs* genes that code for the cellulose synthase complex subunits (Fig. 1*A*). Instead, these missing base pairs code for four adjacent amino acids from the N-domain of the ClpA protein (ClpA_84-87_: QRVI, Fig. 5 *A* and *B*).

The ClpA protein is part of the ClpAPS protease complex, which is responsible for the hydrolysis of misfolded and degraded proteins inside cells (52, 53). The function of ClpA is to bind, unfold, and lead proteins to ClpP, where the hydrolysis process takes place. Under normal conditions, some of the proteins to be degraded are fixed through the specific interaction between the N domain of ClpA and the adaptor protein ClpS, as previously observed in *Escherichia coli* (51, 54). The base pairs deleted from the genome of the evolved strain code for four-amino acids of the ClpS-binding domain of ClpA. In particular, the arginine residue (R_85_) was shown to be highly conserved across species and involved in the interaction between ClpS and ClpA (53).

We thus hypothesized that the in-frame four-amino acid deletion in ClpA limited the ClpS–ClpA interaction without hindering the function of ClpA, thereby contributing to the increased cellulose production by the evolved bacteria (Fig. 5*B*). To test this hypothesis, a *ΔclpS* knockout strain was generated from the native strain through homologous recombination using a pUC19 plasmid backbone (55). Gel electrophoresis and DNA sequencing confirmed the successful deletion of the *clp*S gene in the knockout strain (*SI Appendix*, Fig. S13). Experiments were performed to compare the *ΔclpS* knockout and native strains in terms of cellulose formation in bulk pellicles as well as bacterial growth in liquid culture medium.

The *ΔclpS* knockout strain exhibited enhanced cellulose production of 25% compared to the native strain (Fig. 5*C*). The observed increase in cellulose production by the *ΔclpS* strain suggests a thus far unknown connection between the ClpS–ClpA interaction and cellulose synthesis regulation. ClpS has previously been shown to alter the degrading specificity of the ClpAP complex (54). Our results suggest that such a shift in protein degradation specificity might favor cellulose production in *K. sucrofermentans*. This is in agreement with earlier research on *C. glutamicum*, which showed that a *clpS* deletion can increase the expression of a heterologous protein by 65% (56). In addition to cellulose production, cell growth experiments revealed that the control, evolved, and *ΔclpS* strains had accelerated growth rates compared to the native strain (Fig. 5*D* and *SI Appendix*, Fig. S14).

The fact that the *ΔclpS* knockout strain did not reach the 66% increase in cellulose production observed in the evolved overproducers in this experiment indicates that a complete lack of ClpS–ClpA interaction might not be optimal, or that other factors than the ClpS–ClpA interaction might also contribute to the increased cellulose biosynthesis in the evolved bacteria. Since the control strain showed an accelerated growth rate (Fig. 5*D*) and also produced 19 to 26% more cellulose than the native strain (Figs. 4*D* and 5*C*), we expect the stresses imposed on the bacteria during the mutagenesis treatment steps to account for at least part of the nongenetic contributions to the enhanced cellulose production. We also noticed a 17% increase in cellulose production in sorted native cells compared to native unsorted cells (*SI Appendix*, Fig. S15*A*), further indicating the role of epigenetic factors. Such an increase in cellulose production associated with sorting decreased to 3.6% when the sorting step was applied to the control strain (*SI Appendix*, Fig. S15*B*). These findings highlight the combined importance of genetic diversification, environmental factors, and sorting steps in the directed evolution process to achieve the maximal performance of *K. sucrofermentans* in cellulose production.

## Conclusions

The directed evolution of the microorganism *K. sucrofermentans* in microfluidic droplets allowed for the identification of a bacterial strain for the efficient bio-manufacturing of cellulose as bulk pellicles and engineered living materials. A commonly used fluorescent dye was found to be an effective probe for the high-speed quantification of cellulose produced by single microorganisms inside the microfluidic droplets. Starting with a library of 40,000 randomly mutated variants, four microorganisms were identified as cellulose overproducers at the end of the directed evolution process. When cultivated for 12 d in culture medium, these selected bacteria were able to form pellicles with 54 to 70% more cellulose than the native strain. Using hydrogel inks as hosts for the selected bacteria, we show that the evolved microorganisms can also be 3D printed into complex cellulose-based objects with structural features spanning multiple length scales. Genomic analysis revealed that the cellulose overproducers contained a 12-base pair deletion in the *clpA* gene, which encodes an important binding domain of the protease complex ClpAPS. The link between cellulose overproduction and the specificity of the ClpAPS complex was confirmed by the increased cellulose-forming ability of an engineered strain with dysfunctional protease units. By enabling the evolution of entire microorganisms toward specific functionalities, the high-throughput directed evolution platform shown in this work holds great potential as an identification tool for microbial strains and genotype–phenotype links for bio-manufacturing processes. Moreover, the association between *clp* genes and cellulose overproduction opens new scientific questions and technological avenues for the genetic manipulation of cellulose-forming microorganisms. Beyond the realm of sustainable and living materials, the directed evolution of whole microorganisms might also be an effective strategy to enhance the efficiency of existing and prospective biotechnological processes.

## Materials and Methods

### Cell Culture for BC Production.

*K. sucrofermentans* JCM 9730 (American Type Culture Collection, ATCC 700178) is the cellulose-producing strain used in this study. Their growth medium was composed of 25 g/L D-mannitol (Thermo Fisher Scientific), 5 g/L yeast extract (Sigma-Aldrich), 3 g/L peptone (Sigma-Aldrich), and optionally 15 g/L agar (Sigma-Aldrich) for solid medium. Frozen stocks of *K. sucrofermentans* (−80 °C) were streaked on medium plates to isolate single colonies, which were then inoculated in 5 mL growth medium in 50 mL Falcon tubes Techno Plastic Products AG (TPP). The lid was replaced with a foam plug to allow optimal oxygen availability. Cultures were incubated in static conditions at 28 °C for 8 to 12 d to form BC pellicles at the air–medium interface. For passage experiments (phenotype retention), a sterile inoculation loop was used to rub the BC pellicles and was streaked on solid medium, from which single colonies were picked for subsequent liquid cultures.

### Cell Concentration.

All experiments started from frozen stocks of bacteria. For each stock, serial dilutions from 10^−1^ to 10^−6^ were prepared and plated on solid medium in drops of 5 µL (*n* = 6). CFU were counted at an appropriate dilution and averaged, and the stock concentration was backcalculated.

### Absorbance Measurements.

Absorbance measurements were taken with the Varioscan LUX (Thermo Fisher Scientific) at 600 nm on 200 µL samples in 96-well plates (flat bottom, TPP) unless stated otherwise. Samples were always measured in triplicates, averaged, and blanks were subtracted. Blanks corresponded to medium, with 2 vol% cellulase (Trichoderma reesei ATCC 26921, Sigma-Aldrich) and 35 μg/μL chloramphenicol (Sigma-Aldrich) in case it was also present in the samples.

### UV-C Mutagenesis.

*K. sucrofermentans* from frozen stocks were inoculated in 150 mL of liquid medium for 6 d at 28 °C at 200 rpm. 2 vol% cellulase was added to the culture to digest any cellulose produced. After measuring the absorbance, cells were spun down at 3,275 rcf for 10 min (Z306 Hermle) and resuspended in 0.9% NaCl (VWR) to reach a theoretical absorbance of 1. 10 mL of the cell suspension was added to each Petri dish (diameter Ø of 10 cm, TPP) to be exposed to different UV-C doses without the lid: 0, 0.5, 1, 2, 3, 5, 10, and 100 mJ/cm^2^ (254 nm, UVP Crosslinker CL-3000, AnalytikJena). The plates were then left in the dark for 1 h, spun down, resuspended in enriched medium (2× concentration) with 2 vol% cellulase, and incubated at 28 °C and 200 rpm for either 1 h or 50 h. To store the cultures at −80 °C for further analysis, aliquots with 20 vol% glycerol (Fisher BioReagents) were prepared. To quantify the cell survival, the 1 h cultures were serial diluted and plated on solid medium (5 µL dots, *n* = 6). CFU were counted at the 10^−4^ dilution and compared to the control sample, which was not exposed to UV-C (0 mJ/cm^2^). The 50 h aliquots were used for the directed evolution process, assuming more recovery time was beneficial to increase our chances of finding a strain that grows sufficiently well in our culture medium. The sample exposed to 0 mJ/cm^2^ and recovered for 50 h was used as our control strain for the rest of the study to check the effect of the stresses the cells were exposed to (salt, dark, recovery) on cellulose production.

### Fabrication of Microfluidic Devices.

The photoresist SU-8 (3000 series, MicroChem) was patterned on silicon wafers using standard photolithography methods and used as masters for 1:10 (polydimethylsiloxane (PDMS), Sylgard™ 184, Dow Corning) soft lithography. Briefly, PDMS, after mixing, was poured on the masters, degassed, and cured at 70 °C for 3 to 5 h. After curing, devices were peeled off, and the inlet and outlet holes were punched. Both the emulsifier and sorter were bonded using an air-plasma treatment. Emulsifier devices were bonded to glass slides (Fisherbrand^TH^ Superfrost™) using a 20 s air-plasma treatment at 4*10^−1^ mbar on medium level (Plasma Cleaner PDC-32G), and placed on a hot plate at 90 °C for 1 h to promote further the bonding. Sorters were bonded to a PDMS-coated silicon wafer (spin-coated at 2,400 rpm for 10 s and cured) through an air-plasma treatment for 1 min (Zepto, Diener), placed on a hot plate at 120 °C for 2 h, and finally cut off and bonded to a 50 × 24 × 0.17 mm glass coverslips (Glaswarenfabrik Karl Hecht), using the same air-plasma and heat treatment. To hydrophobize the channels of both devices, a 2 vol% solution of 1H,1H,2H,2H-perfluorooctyltrichlorosilane (Fluorochem) in HFE-7500 (3 M) was flushed through the inlet and then air-dried. For the sorting devices, electrodes were created by inserting a low melting point solder (51In/32.5Bi/16.5Sn; Indium Corporation) at one end of the electrode channel and a wire at the other end. The entire chip was subsequently placed on a 150 °C hotplate for 3 min to melt and reflow the solder across the channel.

### Single-Cell Encapsulation and Incubation.

#### Cell loading.

Cell loading was determined based on the well-known Poisson distribution, according to which the probability (*p*) of a droplet to contain *k* cells depends on the average number of cells per droplet volume λ as follows: $p\left(k,\lambda \right)=\frac{\lambda^{k}e^{-\lambda }}{k!}$. Knowing the droplet size and the frozen stock concentration, we aimed at a λ of 0.1 CFU/droplet, corresponding to a ~9.5% droplet occupancy. This low number was chosen to minimize coencapsulation events (<0.5%). For each encapsulation experiment, cells were thawed from frozen stocks, and their concentration was adjusted to a λ value of 0.1 CFU/droplet (~1.6 M CFU/mL). The occupancy was validated by calculating the percentage of droplets in which cellulose was produced through image analysis (see section *Droplet Image Analysis*).

#### Cell encapsulation.

Cells were encapsulated in 49 μm droplets using a step emulsification PDMS microfluidic device (57, 58). The inner aqueous phase was composed of medium, cells at low concentration (λ ~ 0.1 CFU/droplet), 218 μm FB (Sigma-Aldrich) to specifically stain the cellulose, and 26 μm fluorescein (Sigma-Aldrich) to generate a baseline signal in the droplets. The outer phase comprised 2 wt% 008-FluoroSurfactant in HFE-7500 (RAN Biotechnologies). Both phases were flown at 500 µL/h for 10 min, which enabled the formation of more than a million droplets.

#### Droplet incubation.

Droplets were incubated in hydrophobic glass vials at 28 °C on top of 200 µL HFE-7500 (3 M) in static conditions for 24 h before the sorting process. Vials were horizontally placed to form a monolayer of droplets and thereby ensure that bacteria had equal access to oxygen during incubation.

### Droplet Image Analysis.

Prior to screening in microfluidic devices, droplet sizes, and cellulose content were analyzed using z-stacks of confocal images (TCS SP8, Leica, 30 slices, 3.58 μm each). A 405 nm laser was used to excite the cellulose-specific dye (FB, Sigma-Aldrich), which was detected between 432 nm and 460 nm (HyD detector). An additional PMT transmission detector was used to image the droplets. Using ImageJ (59), FB images were summed, and one of the droplet images was chosen for edge detection. Droplet diameters were estimated using the Hough Transform plugin (UCB Vision Sciences) after image thresholding. For fluorescence quantification in the droplets, the FB sum stack and chosen droplet images were imported to Cell Profiler 4.1.3 (www.cellprofiler.org), a user-friendly software previously reported for droplet image analysis (60). Briefly, droplets were detected as objects in a desirable size range and filtered out if on the edge of the image before measuring the intensity of each object. Results were exported as an Excel file, and all were analyzed and plotted with MATLAB (R2023a, Math Works). Droplets were considered occupied when their measured FB fluorescence exceeded the maximum fluorescence detected on Day 0.

### Droplet Sorting and Screening.

#### Reinjection.

The incubated droplets were reinjected into the droplet sorting device by pressurizing the incubation glass vial at 100 to 150 mbar (LineUP Flow EZ, Fluigent), transferring the droplets from the vial to the inlet of the sorting device via ID/OD 0.86/1.32 mm polyethylene tubing (Scientific Commodities). Oil (HFE-7500, 3 M) was delivered at a flow rate of 6 to 8 µL/min on both sides of the microfluidic channel to increase the spacing between the flowing droplets.

#### Droplet sorting setup.

The incoming droplets were excited using a 405 nm solid-state laser (Omicron Laserage Laserproduckte) beam (expanded, shaped into a line, and focused on the chip using a 20×/0.45 NA Nikon S Plan Fluor ELWD objective). The fluorescence emission of these droplets was first filtered through a 30 μm pinhole and a 480/30 optical filter (Chroma Technology) and was finally captured using a PMT (H10722-20; Hamamatsu Photonics). The voltage signal from the PMT was sampled at a rate of 100,000 samples per second by an FPGA (NI PXI-7842R; National Instruments) running a custom LabVIEW code. The code registered the signal from incoming droplets and, based on a user-defined threshold, issued a series of tunable pulses (25 to 35 pulses at a pulse frequency of 40 kHz, corresponding to a train length of 0.6 to 0.8 ms) that were fed to the chip via high-voltage amplifier (Trek 632B, Advanced Energy) for droplet sorting (900 V final pulse voltage).

#### Mutant library sorting.

The added fluorescein in the droplets provided a fluorescence baseline to detect all droplets and was adjusted to emit a signal corresponding to 0.05 V. A total of 430,000 droplets were screened in less than 10 min, of which 500 were sorted using a threshold of 2.75 V. Those droplets were collected in a sterile Eppendorf tube. After adding medium and demulsifying, the droplets were plated onto solid medium. Five evolved single colonies were then arbitrarily picked and grown in liquid medium with 2 vol% cellulase, and aliquots with 20 vol% glycerol (Fisher BioReagents) were frozen for further analysis (Ev1 to Ev5).

#### Droplet screening.

To compare the native, control, and evolved variants, the fluorescein baseline was adjusted to 0.1 V across all the measurements. 15,000 droplets of each variant were screened. The screening data were fitted to a Gaussian mixture distribution model with three components and baseline-adjusted using the lowest peak’s mean (corresponding to the fluorescein signal) using the following formula: $\frac{data\;*\;0.1}{\mathrm{fluoresce in}\;m\mathrm{e an}\;\mathrm{signal}}$. The middle mean value (fluorescence signal from the FB) reporting the cellulose production in the majority population was used for comparison among the native, control, and evolved strains.

### BC Dry Weight.

To quantify the amount of BC produced by the evolved strains compared to the native and control (0 mJ/cm^2^) strains, triplicates of BC pellicles were grown, as previously explained. After 12 d, the pellicles were washed 3 times with 0.1 M NaOH (Fisher Scientific) at 60 °C in a water bath over a period of 24 h and then brought back to neutral pH washing 3 times with MilliQ water (NANOpure Diamond, Barnstead). They were then dried in a 60 °C oven for 48 h on Teflon films (McMater-Carr) to avoid sticking and stored under vacuum until analyzed. Dried BC pellicles were weighed with a precision balance (UMT2 Microbalance, Mettler Toledo). TGA (Discovery TGA 5500, TA Instruments) was also performed on the dried cellulose pellicles by increasing the temperature to 650 °C at a rate of 10 °C/min. During TGA, an isotherm of 15 min at 120 °C was applied to remove all the humidity contained in the samples. Weight loss was then calculated between the end of the 120 °C isotherm and the end of the program at 650 °C.

### 3D Printing.

#### Ink preparation.

The ink was composed of 1.5 wt% sodium hyaluronate (BulkSupplements), 1.5 wt% κ-carrageenan (Acros Organics), and 1.5 wt% fumed silica (WDK V15, Wacker Chemie) in the standard medium used for *K. sucrofermentans* described above. The ink was prepared following a previously published protocol (10). UV-C was used to sterilize all powders before mixing. 218 μm of Fluorescent Brightener (Sigma-Aldrich) was also added to stain the cellulose produced in the ink. For each experiment, a new batch of ink was prepared and separated in three equal amounts, in which 0.36 M CFU/g of bacteria were added from frozen stocks. For controls, the same volume of sterile medium was added to the ink (900 µL). The final inks were loaded into 10 mL syringes and kept at 4 °C until 3D printing on the same day.

#### Ink rheology.

Rheological measurements of the inks were performed under oscillatory and steady-shear conditions using a sandblasted parallel plate geometry (PP25-S, Anton Paar) at 25 °C (MCR 302 compact rheometer, Anton Paar). The storage and loss moduli (*G’* and *G’’*) were measured via oscillatory rheology by applying a shear strain amplitude increasing logarithmically from 0.01 to 100% at a constant frequency of 1 s^−1^. Steady-shear flow curves were then recorded under strain-controlled conditions by ramping the shear rate logarithmically from 0.01 to 100 s^−1^ and then back from 100 to 0.01 s^−1^.

#### Direct ink writing (DIW).

All structures were 3D printed via the DIW technique using a 10 mL syringe, a 0.84 mm needle, and a layer height of 0.7 mm. For the quantification of the cellulose content grown within the ink, one-layer disks of 12 mm diameter were printed directly on Petri dishes. Immediately after completion of the print, the disks were covered with coverslips to ensure that oxygen was only available from the sides. Each Petri dish was then sealed with parafilm and imaged as is with a confocal microscope (TCS SP8, Leica) after a day of incubation at 28 °C and 85% relative humidity in static conditions. For each full disk, a 15-slice stack of 750 μm total was imaged (excitation: 405 nm; emission: 432 to 460 nm) and stitched together using Leica software.

#### Image analysis.

All the image analysis was performed using ImageJ (59). Slices of the stacks were averaged, and the radial profile of each disk was plotted. Data were then smoothed and plotted with MATLAB (R2023a, Math Works).

### Genome Sequencing, Assembly, and Analysis.

Genome sequencing was performed by MicrobesNG, United Kingdom. The *K. sucrofermentans* JCM 9730 (ATCC 700178) reference genome was sequenced with an Illumina NovaSeq 6000 (Illumina, San Diego, USA) using a 250 bp paired-end protocol, as well as with GridION (Oxford Nanopore Technologies, UK) to obtain long reads. Strains selected from the directed evolution process were sequenced with Illumina NovaSeq 6000 (Illumina, San Diego, USA) only.

Reference genome assembly combined both long and short reads using Unicycler version 0.4.0 (61), and annotation was performed with Prokka 1.13 (62). To quantify the quality of the assembly, coverage statistics were calculated using the short read data using samtools 1.3.1 (63). The obtained statistics are shown in *SI Appendix*, Table S1. Mutations were detected using breseq 0.35.1 (49), aligning the Illumina paired-end reads to the annotated reference genome. Bioinformatic analysis of specific genes and operons was performed with BioPython (64).

### Knockout Generation.

*K. sucrofermentans* knockout was produced by transforming cells with a pUC19 plasmid, which does not replicate inside this bacterium. Further, the pUC19 plasmid was engineered to contain 500 bp regions of homology to the genome around the *clpS* sequence, designed to remove the start codon of *clpS* and disrupt expression (*SI Appendix*, Fig. S13*B*). The homology arms were placed on each side of a *cat* chloramphenicol resistance cassette to create plasmid pUC19-clpS-KO (*SI Appendix*, Fig. S13*A*) through Gibson assembly (65) and cloning in *E. coli* DH5a cells. DNA oligos were obtained from Integrated DNA Technologies (Belgium).

*K. sucrofermentans* cells were made electrocompetent by growing a culture to an absorbance of 0.8 at 600 nm, before centrifuging at 3,000 g for 10 min and washing the pellets three times with 10% glycerol (Fisher BioReagents). Cells were then concentrated and electroporated with pUC19-clpS-KO. Following overnight recovery in medium supplemented with 2 vol% cellulase at 28 °C in shaking conditions (200 rpm), cells were plated onto 25 µg/mL chloramphenicol to select for colonies containing the *cat* cassette. Colonies were screened by colony PCR with GoTaq polymerase (M7423, Promega), using verification primers designed around the insertion site in *clpS* on the genome, *clpS_checkF*: CGAGCACCGCCTGCTCCACCG and *clpS_checkR*: TCGCGACGGGGGCGTTAAGATG. PCR products were run in 1.5% agarose (Ultrapure Agarose, Thermo Fisher Scientific) with gel electrophoresis in Tris-Acetate-EDTA buffer (Fisher Bioreagents). Sybr Safe (Thermo Fisher Scientific) DNA stain was added initially to the gel, and after electrophoresis alongside a 1 kb Plus DNA Ladder (Thermo Fisher Scientific), imaging was performed using a ChemiDoc™ MP imaging system (BioRad, *SI Appendix*, Fig. S13*C*). Further verification was performed by Sanger Sequencing the PCR products with the verification primers (Microsynth, Switzerland), which in each case found the expected sequence.

### Growth Curve.

Each tested strain was thawed, spun down at 3,000 rcf for 10 min (5417R, Eppendorf), resuspended in 1 mL of fresh medium, and their absorbance at 600 nm adjusted to 0.005 by dilution. 2 vol% of cellulase was added to each culture to prevent the formation of a cellulose pellicle. 1 mL of each culture was then transferred to 24-well plates (flat bottom, TPP) in triplicates, and the well plates were covered with a breathable film (BREATHseal™, Greiner Bio-One) to avoid contamination between wells. The well plates were incubated at 28 °C and 85% relative humidity in shaking conditions (200 rpm). Every day, the breathable film was removed for the measurement, the absorbance at 600 nm of each well was measured, triplicates were averaged, and blanks composed of medium and 2 vol% cellulase were subtracted from the measurement. The breathable film was changed, and the plates were incubated until the next measurement.

### Statistical Analysis.

The significance of the increased cellulose production was evaluated with a one-way ANOVA (MATLAB R2023a, Math Works), followed by a pairwise comparison if the results showed a statistically significant difference between the groups (*P* < 0.05). A Bonferroni correction was applied to compensate for the effects of multiple comparisons.

## Supplementary Material

Appendix 01 (PDF)

## Data Availability

Genome sequences of the *K. sucrofermentans* native JCM 9730 (SAMN37928908), evolved JML 2321 (SAMN37928909), and *ΔclpS* knockout JML KO 23 (SAMN37928910) strains were uploaded to NCBI (https://www.ncbi.nlm.nih.gov) (50).
