# Supplementary material for: Directed evolution of material-producing microorganisms
Source: Proc Natl Acad Sci U S A. 2024 Jul 23;121(31):e2403585121. doi: 10.1073/pnas.2403585121 (PMC11295069; doi:10.1073/pnas.2403585121)
Supplement: Supplementary file 1 — Appendix 01 (PDF) [file pnas.2403585121.sapp.pdf]

Supporting Information for

Directed Evolution of Material-producing Microorganisms

Julie M. Laurent,[1] Ankit Jain,[2] Anton Kan,[1*] Mathias Steinacher,[1] Nadia Enrriquez Casimiro,[1] Stavros Stavrakis,[2] Andrew J. deMello,[2] André R. Studart [1 *]

[1] Complex Materials, Department of Materials, ETH Zürich, 8093 Zürich, Switzerland

[2] Institute for Chemical and Bioengineering, Department of Chemistry and Applied Biosciences, ETH Zürich, 8093 Zurich, Switzerland

* corresponding authors: andre.studart@mat.ethz.ch, anton.kan@mat.ethz.ch

This PDF file includes:

Supporting text
Figures S1 to S15
Table S1
SI References

Supporting text

Direct ink writing of bacteria-laden gels

Cellulose-producing bacteria were loaded in biocompatible gels and printed into complex-shaped geometries using the Direct Ink Writing (DIW) techniques. Oscillatory rheology experiments confirmed that both cell-laden and cell-free gels display an elastic response at low shear stresses and become a viscous fluid above a well-defined yield stress of 200 Pa (Figure S9c and Figure S10, Supporting Information). These properties fulfill the rheological requirements for 3D printing via the extrusion-based DIW technique (Figure S9a).

To gain insights into the effect of the bacterial strain on cellulose formation within the gel, we printed 12-mm discs containing the cellulose-binding dye, loaded them with either the evolved or native strains, and imaged them using confocal microscopy (Figure S9d-h). Fluorescence emitted by the cellulose-binding dye was taken as a proxy for the local concentration of cellulose produced by the bacteria. To restrict oxygen supply to the edges of the sample, the printed discs remained enclosed between a Petri dish and a cover slip during the incubation and imaging processes (Figure S9d).

Confocal images of the printed disks showed cellulose production predominantly along the edges of both types of samples next to the air-water interface. Notably, the evolved bacteria produced more cellulose than the native bacteria, leading to the formation of a cellulose-rich narrower ring at the edge of the disk (Figure S9f and Figure S11, Supporting Information). This contrasts with the broader and less intense fluorescent cellulose ring in the samples containing the native microorganisms (Figure 4e and Figure S11, Supporting Information). Radially integrated fluorescence measurements of the samples provide clear evidence of the distinct cellulose patterns created by the two bacterial strains (Figure S9g,h). The ability to overproduce cellulose in a viscoelastic gel makes the evolved strain suitable for the 3D printing of engineered living materials with tunable architecture across multiple length scales.

Figures

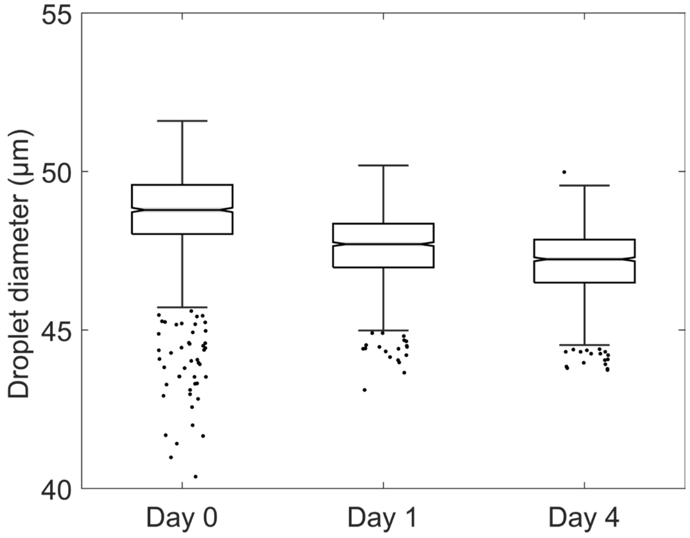

Figure S1. Diameters of cell-laden droplets (λ ~0.1 CFU/droplet) produced with the parallelized step emulsification microfluidic device. Statistical analysis led to the following mean droplet diameters (Ø) and polydispersity indices (PDI): Day 0: Ø = 48.8 µm, PDI = $1.1*10^{-3}$; Day 1: Ø = 47.7 µm, PDI = $4.9*10^{-4}$; Day 4: Ø = 47.2 µm, PDI = $4.8*10^{-4}$ (n > 1000).

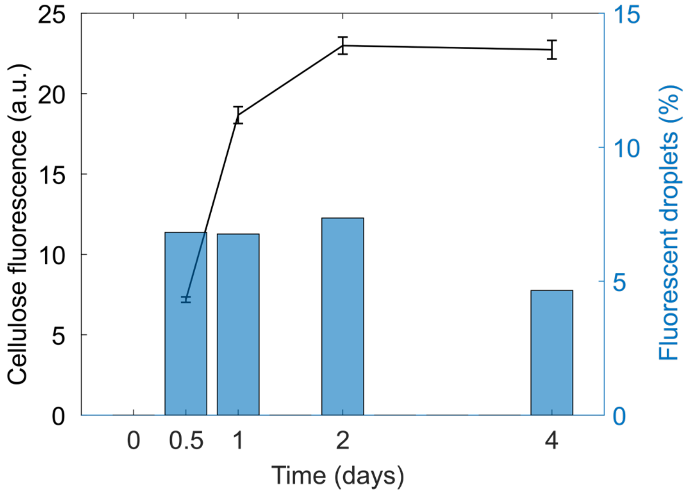

Figure S2. Evolution of the quantity of fluorescently labeled cellulose in droplets over time. Confocal microscopy images were analyzed, and the cellulose-correlated fluorescence in individual droplets was measured (n ~1200 droplets). The majority of the cellulose was produced within the first day of droplet incubation, with a maximum fluorescence reached after two days of incubation (left axis). Error bars correspond to the standard error of the mean. About 7% of droplets contained cellulose-producing bacteria, which is close to the 9.5% value expected from Poisson statistics for a cell concentration λ of 0.1 CFU/droplet (right axis).

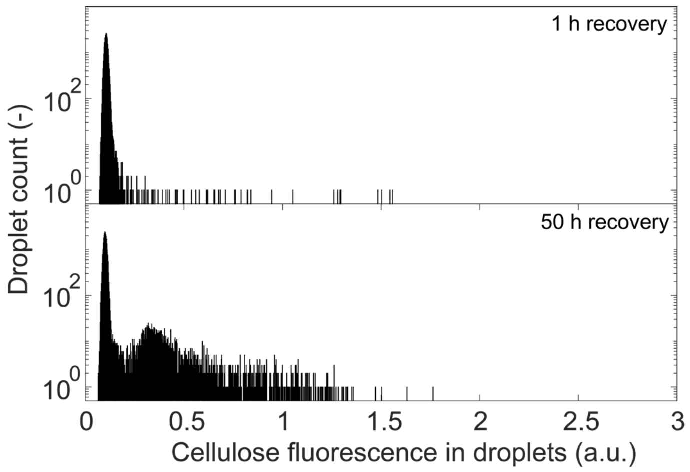

Figure S3. Histogram of cellulose fluorescence signals in droplets containing single cells after mutagenesis (10 mJ/cm^2) with 1 h or 50 h of recovery (λ ~0.1 CFU/droplet, 28’000 droplet events). Screening was performed after incubation of the droplets at 28°C for 24 h. Mutants recovered after 50 h show more cellulose than in the 1 h-recovered strain, as evidenced by the highly fluorescent droplets detected for the former case. Using the library of 50 h-recovered mutants is expected to increase the chances of finding a suitable candidate for growth under laboratory conditions.

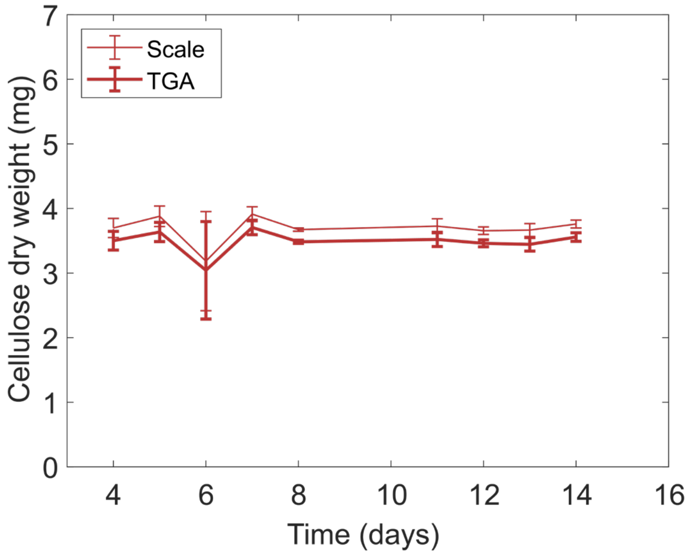

Figure S4. Weight of bacterial cellulose (BC) pellicles formed by the native strain over time. BC pellicles with a diameter of 3 cm were grown from single colonies under static conditions in 5 mL growth medium at 28°C. After growing for different amounts of days, the pellicles formed at the air-water interface were washed and air-dried to determine the variability in cellulose dry weight resulting from slightly different inoculation quantities. Weights were measured with both a laboratory scale and extracted from TGA for each washed and air-dried sample (n = 3). After 8 days, the weight of the cellulose stabilized. Error bars represent standard deviations.

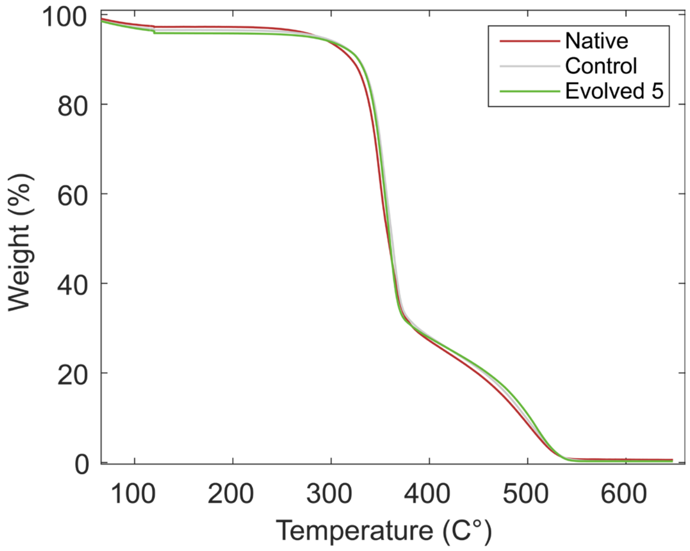

Figure S5. Representative thermogravimetric analysis (TGA) curves of bacterial cellulose produced by the native (red), control (grey), and evolved (green) strains after 12 days of incubation of single colonies at 28°C in static conditions. The washed and air-dried samples were heated up to 120°C (10°C/min), then an isothermal step was set for 15 min to ensure all the water evaporated, and finally, samples were thermally degraded to 650°C (at 10°C/min). Weight loss was then calculated between the end of the isotherm and the end of the program.

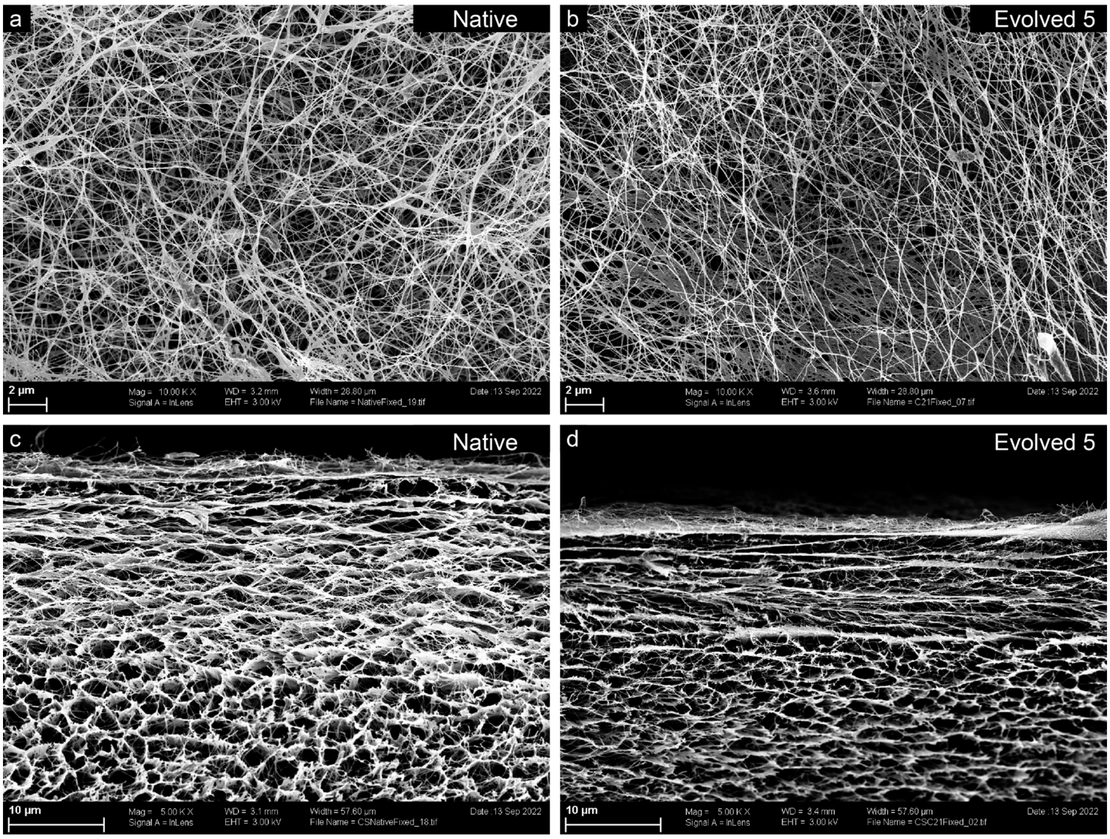

Figure S6. Scanning electron microscopy of freeze-dried bacterial cellulose pellicles produced by the native (**a, c**) and evolved 5 (**b, d**) strains after 12 days of incubation at 28°C in static conditions. **a** and **b** originate from the bottom fibers of the pellicle. **c** and **d** show the cross-section of the pellicle. The long fibers produced by both strains appear similar.

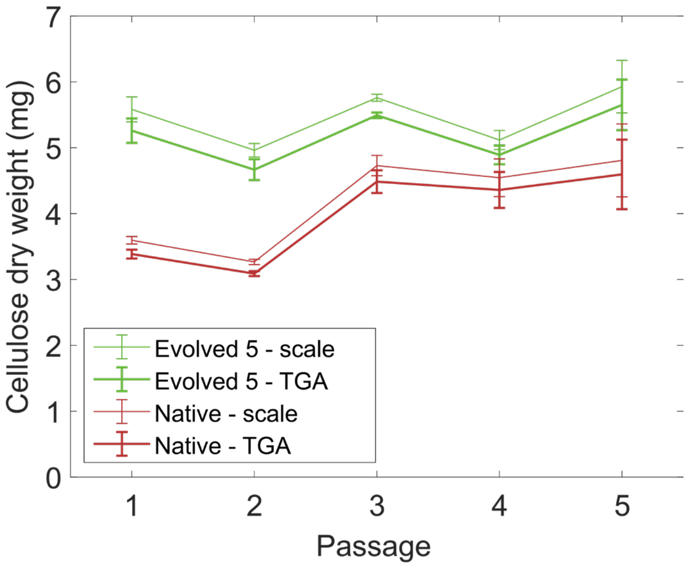

Figure S7. Weight of bacterial cellulose (BC) pellicles grown after increasing number of passages. BC pellicles with a diameter of 3 cm were grown under static conditions in 5 mL growth medium at 28°C from single colonies for 8-12 days. The single colonies were obtained from previous pellicles over 5 generations. The weight was measured both with a laboratory scale and extracted from TGA for each washed and air-dried sample (n = 3). The evolved strain (green) shows a stable phenotype of increased cellulose production. The native strain (red) shows a spontaneous increase in cellulose production after the third generation, but always less than the evolved strain. Error bars represent the standard deviation.

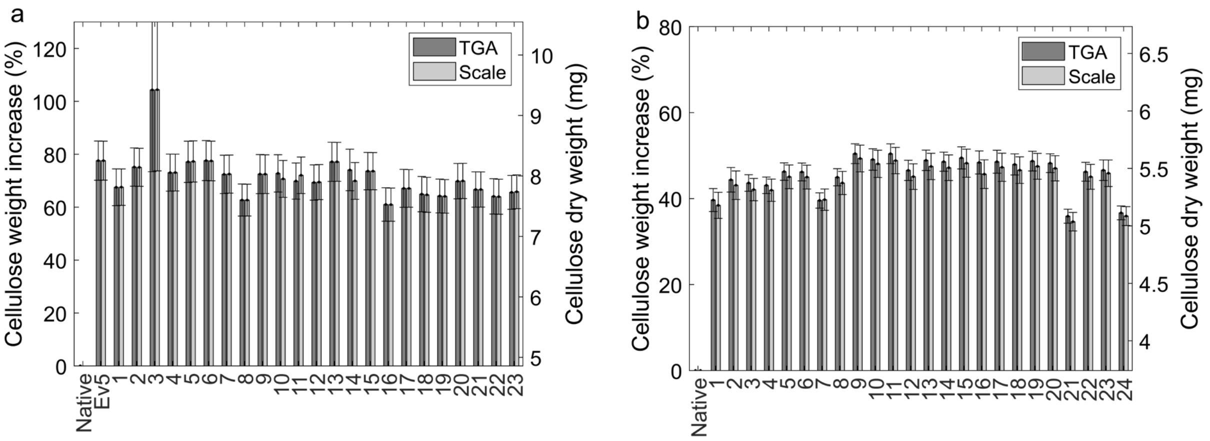

Figure S8. Second round of directed evolution of *K. sucrofermentans*. The evolved strain from the first round of directed evolution (Ev5) was mutated (10 mJ/cm^2), encapsulated (λ ~0.1 CFU/droplet), and sorted with two different voltage thresholds: the same as the first round 2.75V (**a**) and a higher threshold of 3.60V (**b**). Bacterial cellulose pellicles with a diameter of 3 cm were grown under static conditions for 12 days from single colonies of all the selected strains of this second round (5 mL growth medium, 28°C, static). The weight was measured both with a laboratory scale and extracted from TGA for each washed and air-dried sample (n = 3). All selected strains show a phenotype comparable to Ev5 but no further increase in cellulose production. Error bars represent the propagated errors for the percentage data.

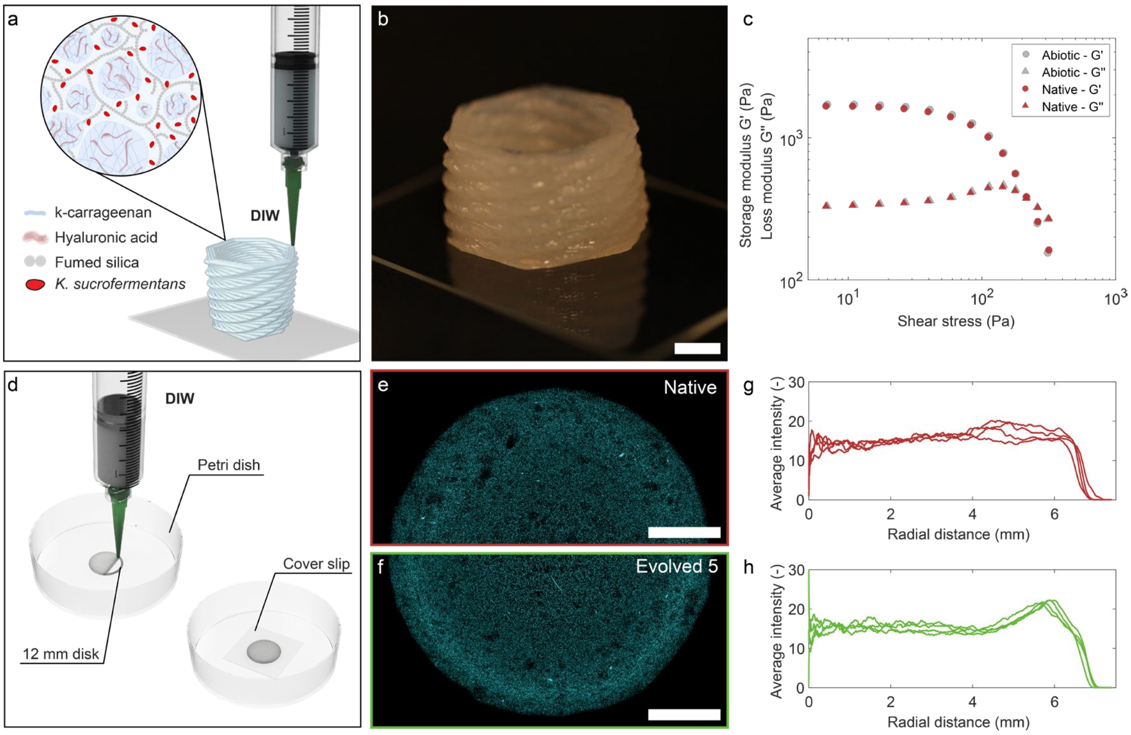

Figure S9. 3D printed engineered living material. a. Scheme of the Direct Ink Writing (DIW) process used to 3D print a complex-shaped object from a gel loaded with *K. sucrofermentans*. The gel contained 1.5 wt% κ-carrageenan, 1.5 wt% hyaluronic acid, 1.5 wt% fumed silica, growth medium, and 0.36 M CFU/g bacteria. **b.** Image of the centimeter-scale engineered living object obtained by 3D printing the bacteria-laden ink. Scale bar: 1 cm. **c.** Rheological properties of the ink with no bacteria (grey) or with the native bacteria (red). The crossover between the storage (*G'*) and loss (*G'*) moduli occurs at a yield stress of 200 Pa for both inks. **d.** Schematics depicting the 3D-printed monolayer disks used to measure cellulose formation within the ink. A coverslip was utilized to limit oxygen access to the sides of the sample. **e-f.** Representative stitched confocal images of fluorescently labeled bacterial cellulose in the 3D-printed disks containing (e) the native or (f) the evolved (Ev5) strains after 1 day of incubation. Scale bars: 3 mm. **g-h.** Radial integration of the cellulose fluorescence over the whole disks containing the evolved and native strains (*n* = 4).

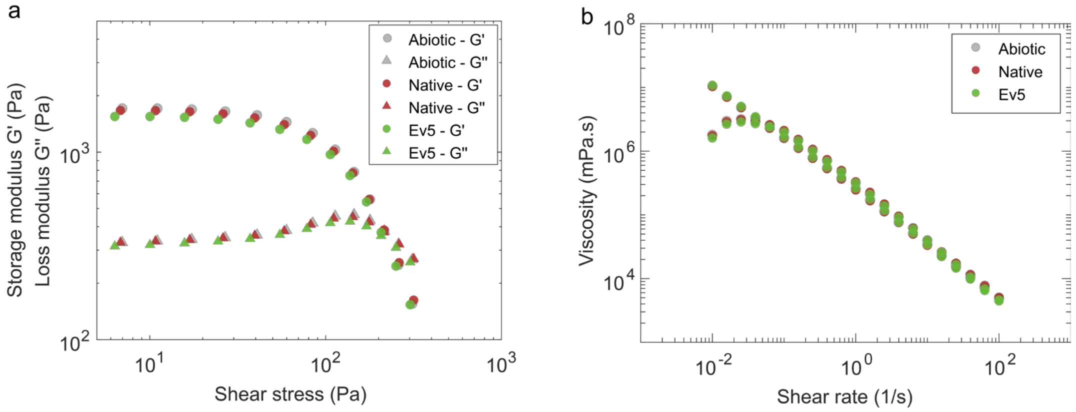

Figure S10. Rheological properties of the cell-laden gel used for 3D printing. **a.** Oscillatory rheology of gels containing native or evolved (Ev5) strains compared to the cell-free (abiotic) ink. The storage (*G'*) and loss (*G''*) moduli of the gels were measured for applied shear strains that varied from 0.01 to 100%. The gel exhibits an elastic response at low shear stresses (*G'* > *G''*) and becomes predominantly fluid (*G''* > *G'*) above 200 Pa. **b.** Steady-shear flow curves were obtained by applying a shear rate that increased from 0.01 to 100 s^{-1}. The gels show a shear-thinning behavior that is suitable for 3D printing via Direct Ink Writing. Grey: abiotic gel; red: gel with native bacteria (0.36 M CFU/g bacteria); green: gel with evolved bacteria 5 (0.36 M CFU/g bacteria).

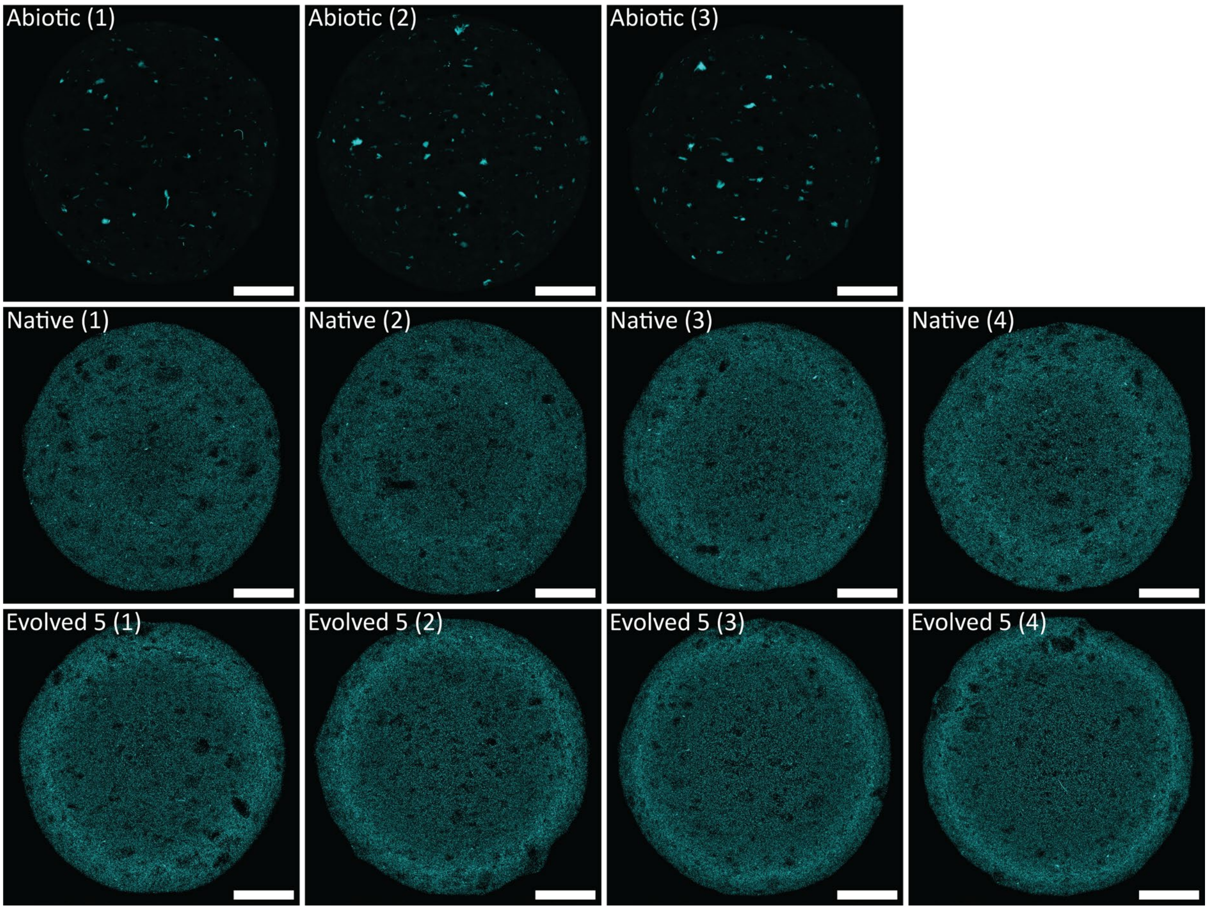

Figure S11. Confocal images of 3D-printed bacteria-laden disks (0.36 M CFU/g bacteria) after 1 day of incubation at 28°C (*n* = 4). The 12-mm disks are covered with a cover slip to ensure oxygen is only available through the sides. Fluorescently labeled cellulose is imaged using excitation and emission wavelengths of 405 nm and 432-460 nm, respectively. Images show that both strains produce more cellulose at the edge of the disk, at the air-water interface. The evolved strain 5, however, produces more cellulose in a narrower ring, whereas the ring where the native strain produces more cellulose is broader and less intense. Scale bars: 3 mm.

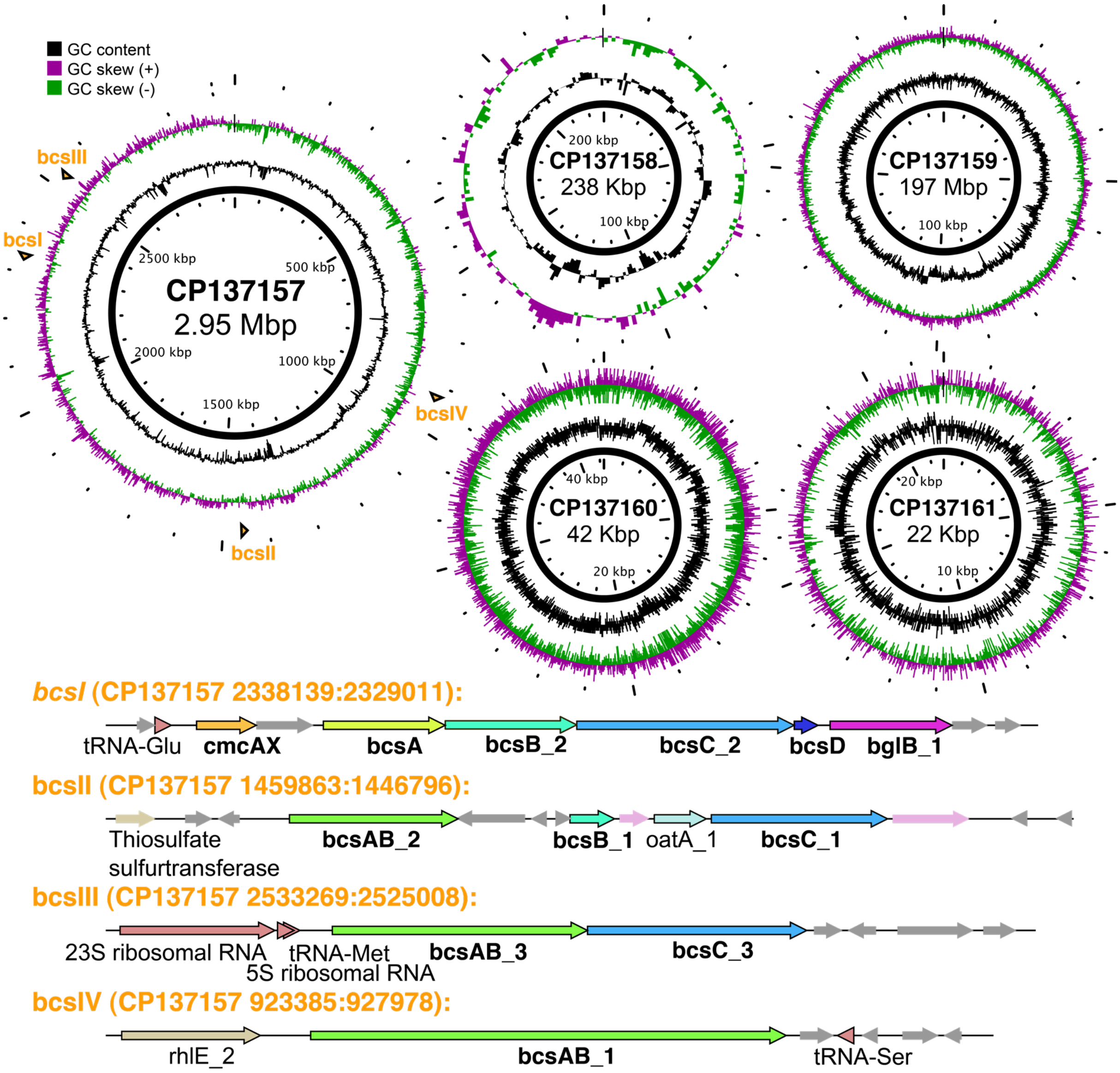

Figure S12. *Komagataeibacter sucrofermentans* assembled genome (NCBI accession SAMN37928908). The genomic DNA (CP137157) is 2.95 Mbp and contains 4 operons containing genes coding for bacterial cellulose synthase subunits. *bcsI* (2338139:2329011) contains *bcsA*, *bcsB_2*, *bcsC_2* (also annotated as *acsC_2*), and *bcsD* (*acsD*) genes. *bcsII* (1459863:1446796) contains *bcsAB_2* (*acsAB_2*), *bcsB_1*, and *bcsC_1* (*acsC_1*) genes. *bcs III* (2533269:2525008) contains *bcsAB_3 (acsAB_3)*, and *bcsC_3 (acsC_3)* genes. *bcsIV* (923385:927978) contains only the *bcsAB_1* (*acsAB_1*) gene. Four additional plasmids are present in this strain: p1 CP137158 (238 Kbp), p2 CP137159 (197 Kbp), p3 CP137160 (42 Kbp), and p4 CP137161 (22 Kbp).

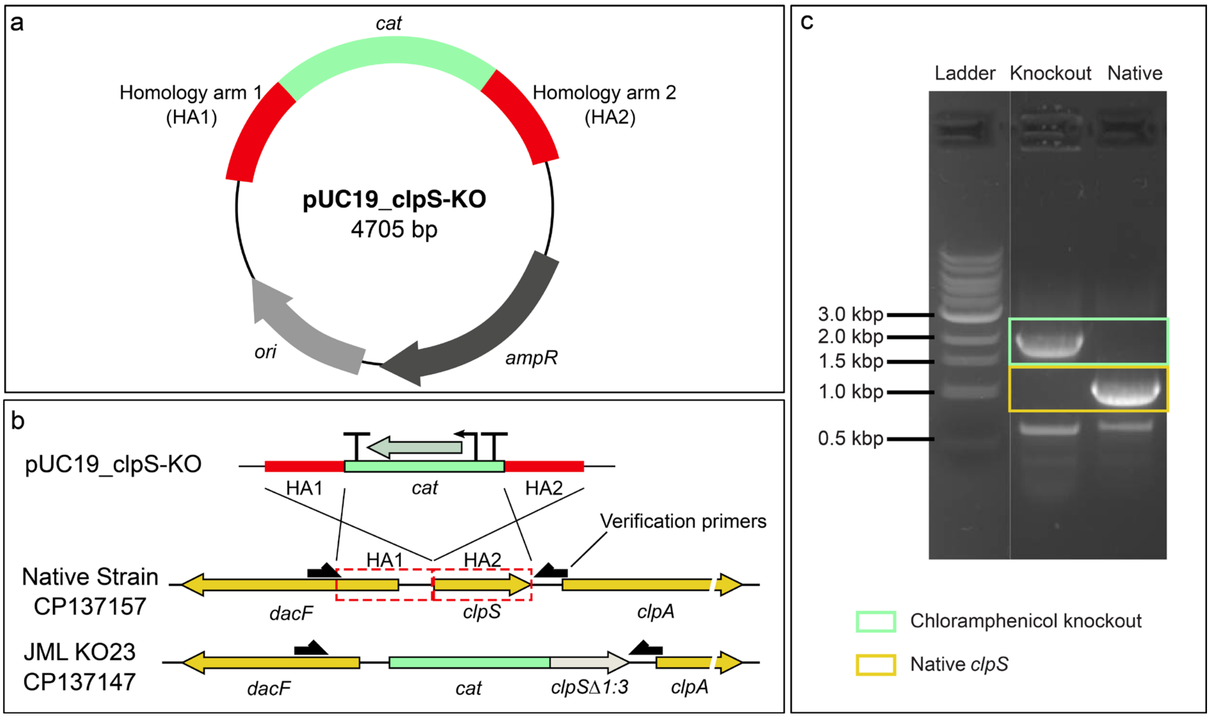

Figure S13. *ΔclpS* knockout generation. **a.** pUC19-clpS-KO plasmid engineered with a pUC19 ampicillin-resistant backbone. 500 bp regions of homology to the *K. sucrofermentans* native genome were designed around the *clpS* sequence (HA1 and HA2). Between the homology arms, a *cat* chloramphenicol resistance cassette was inserted. **b.** pUC19-clpS-KO is designed to remove the start codon of *clpS* and disrupt expression. Verification primers *clpS_checkF* and *clpS_checkR* were designed to confirm the knockout with PCR and sequencing (black arrows). **c.** Gel electrophoresis of PCR amplicons around *clpS* in *ΔclpS* knockout and native strains. The 2 Kpb band in the *ΔclpS* knockout strain confirms the insertion of a chloramphenicol resistance gene into the *clpS* sequence. The 1 Kbp band in the native strain shows the native *clpS* amplicon.

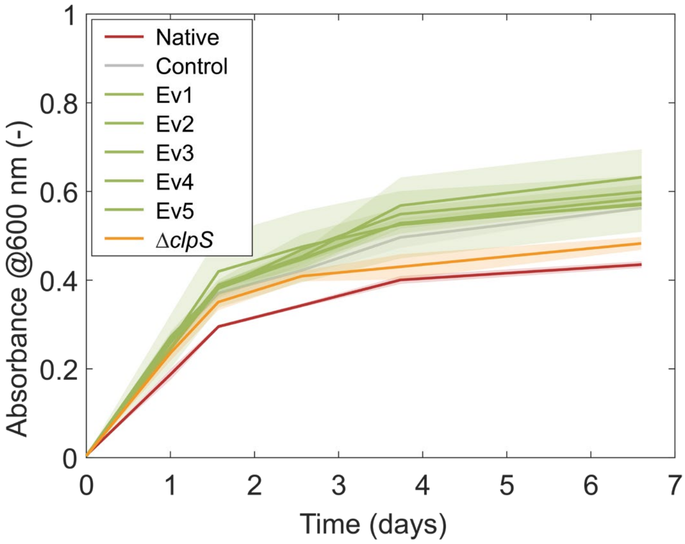

Figure S14. Bacterial growth curves of the different strains in the presence of 2 vol% cellulase in shaking conditions (200 rpm, 28°C, 85% relative humidity). To innoculate similar amounts of bacteria, the absorbance of the different samples was adjusted to 0.005 at 600 nm. Shaded areas around the curves represent the standard deviation (n = 3).

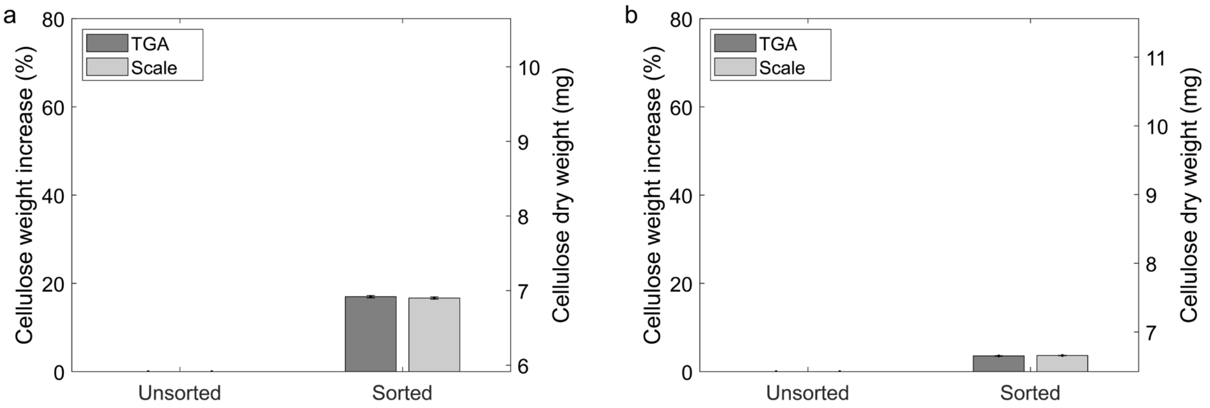

Figure S15. Weight of bacterial cellulose (BC) pellicles obtained from sorted and unsorted strains. The native (**a**) and control (**b**) bacteria were encapsulated using a cell suspension with concentration λ ~0.1 CFU/droplet and afterward sorted in the microfluidic device utilizing a PMT voltage of 2.75 V. Pellicles with a diameter of 3 cm were grown from single colonies of native unsorted and sorted samples (**a**) or control unsorted and sorted samples (**b**) under static conditions for 12 days in 5 mL growth medium at 28°C. Weights were measured both with a laboratory scale and extracted from TGA for each washed and air-dried sample. Increases in cellulose production of 17% and 3.6% were observed when the native and control bacteria were sorted, respectively. Error bars represent the propagated errors for the percentage data (n = 3).

Table

Table S1. Mean Illumina sequencing depths. To verify the quality of the sequencing, the mean sequencing depth was determined for each piece of DNA in the long-read reference genome and in each Illumina sequencing run, quantifying the mean number of reads for each nucleotide position.

	Reference Genome (Native)	Native	Control	Ev1	Ev2	Ev3	Ev4	Ev5	Sorted Native	Sorted Control
**Genome**	110.1	105.2	68.9	58.1	60.7	83.8	56.9	48.9	77.2	55.9
**Plasmid p1**	102.5	103.2	58.1	38.8	40.9	54.1	68.8	60.1	74.4	44.2
**Plasmid p2**	171.5	101.0	56.7	46.3	45.6	58.1	73.1	60.3	82.3	47.1
**Plasmid p3**	89.70	94.40	17.9	12.7	16.4	20.4	19.1	13.8	22.9	19.0
**Plasmid p4**	91.60	91.80	23.2	13.2	27.0	25.7	23.6	17.6	31.1	21.9

SI References

1 Kokkinis, D., Schaffner, M. & Studart, A. R. Multimaterial magnetically assisted 3D printing of composite materials. *Nat Commun* **6**, 8643, doi:10.1038/ncomms9643 (2015).

2 Smay, J. E., Cesarano, J. & Lewis, J. A. Colloidal inks for directed assembly of 3-D periodic structures. *Langmuir* **18**, 5429-5437 (2002).
